# Deep learning–assisted malaria microscopy with sensitivity-aware threshold optimization

**DOI:** 10.3389/fmed.2026.1911275

**Published:** 2026-08-26

**Authors:** Fatma Dehbi, Reda Mohamed Hamou, Menaouer Brahami, Sahmoud Shaaban, Abdelhamid Ghoul, Ameur Latreche, Selman Djeffal

**Affiliations:** 1 LABAB Laboratory, Computer Systems Engineering Department, National Polytechnic School of Oran, Oran, Algeria; 2 GeCoDe Laboratory, Computer Science Department, Dr. Tahar Moulay University, Saida, Algeria; 3 Data Science Application and Research Center (VEBIM), Fatih Sultan Mehmet Vakif University, Istanbul, Türkiye; 4 Automatic Control, Department of Computer Science, Electrical and Space Engineering, Luleå University of Technology, Luleå, Sweden; 5 Department of Biomedical Physiology and Kinesiology, Simon Fraser University, Burnaby, BC, Canada; 6 National Polytechnic School of Constantine, Constantine, Algeria

**Keywords:** convolutional neural networks, deep learning, explainable artificial intelligence, malaria detection, medical image classification, microscopy image analysis, sensitivity optimization

## Abstract

Malaria microscopy becomes clinically risky when parasitized erythrocytes are missed, even when a classifier reports high overall accuracy. This study presents an artificial-intelligence-assisted malaria microscopy framework that explicitly optimizes the diagnostic operating point for sensitivity-aware screening. A balanced set of 27,558 National Institutes of Health/National Library of Medicine (NIH/NLM) thin-smear cell images was divided by stratified image-level sampling in the ratio 70:15:15 for training, validation, and independent testing. Two custom convolutional neural networks (CNNs) with complementary capacity–regularization profiles and an equal-weight score-level ensemble were evaluated. Training-only online augmentation, fixed input resizing, validation-only threshold selection, confidence intervals, and error analysis were incorporated to improve reproducibility. At the conventional threshold, the compact CNN achieved the highest accuracy (95.26%). After optimizing the operating point with the recall-weighted *F*_2_ objective, the deeper CNN provided the strongest screening-oriented result on the held-out test set, reaching 97.05% sensitivity (95% confidence interval: 96.23%–97.70%), a 96.01% *F*_2_-score, a 2.95% false-negative rate, and a receiver-operating-characteristic area under the curve of 0.9876 (95% confidence interval: 0.9842–0.9910). It reduced missed parasitized cells from 177 to 61 relative to its default threshold. The public release does not provide patient identifiers; therefore, the reported split is image-level rather than confirmed to be patient-level independent, and external multicenter validation remains necessary before clinical deployment. The main finding is that the highest-accuracy classifier is not necessarily the most appropriate sensitivity-oriented screening configuration.

## 1 Introduction

Malaria remains a major global health burden. The World Health Organization estimated 282 million cases and 610,000 deaths in 2024, underscoring the continuing need for timely diagnosis and sustained surveillance (1). Light microscopy remains central to malaria diagnosis, but its performance can be influenced by parasite density, staining quality, field selection, workload, and reader experience. Automated image analysis is therefore most appropriately considered as a decision-support tool that can assist, rather than replace, expert microscopy.

Recent deep-learning systems have achieved strong discrimination between parasitized and uninfected erythrocytes, yet a high classification accuracy does not by itself determine whether a model is suitable for screening. In a screening setting, missed parasitized cells may be more consequential than additional false-positive reviews. The operating threshold, probability calibration, uncertainty, and transfer of the decision rule across microscopes and clinical sites therefore require separate consideration. This distinction is especially important when models are evaluated on curated public image collections whose score distributions may differ from those encountered in routine practice.

The present study investigates this decision-policy problem using two custom convolutional neural networks (CNNs) with deliberately different capacity and regularization profiles, together with a fixed equal-weight score ensemble. The models are evaluated at the conventional threshold and at thresholds selected exclusively from validation data by maximizing the recall-weighted *F*_2_ measure. The study additionally reports confidence intervals and paired tests for threshold-induced sensitivity changes, clearly distinguishes threshold optimization from probability calibration, and discusses image-level splitting, local calibration, threshold transferability, and external validation. The objective is not to claim a universally optimal malaria classifier, but to determine how model architecture and operating-point selection interact when sensitivity is prioritized.

## 2 Related work

Early work in medical-image analysis established the broader methodological basis for learning discriminative representations directly from images. LeCun et al. (2) described the general deep-learning paradigm in which hierarchical feature representations are learned from data rather than prescribed manually. Litjens et al. (3) subsequently reviewed the rapid adoption of deep learning across medical-image analysis and highlighted both its performance potential and its dependence on appropriate validation. Shen et al. (4) similarly emphasized that medical-image applications require not only powerful representation learning but also careful task formulation and evaluation. Simonyan and Zisserman's (5) VGG architecture demonstrated the benefit of increasing convolutional depth with small kernels. He et al. (6) introduced residual learning to enable substantially deeper networks while alleviating optimization difficulties. Szegedy et al. (7) refined multi-scale feature extraction through the Inception family. Tan and Le (8) proposed EfficientNet, showing that depth, width, and input resolution can be scaled in a coordinated manner to improve efficiency. Dosovitskiy et al. (9) later showed that transformer-based image representations can provide an alternative to conventional convolutional hierarchies. These architectural advances provide useful context for malaria-image classification, but they do not by themselves determine how a continuous model score should be converted into a screening decision.

For malaria microscopy specifically, Rajaraman et al. (10) demonstrated that features extracted from pre-trained CNNs can accurately separate parasitized and uninfected erythrocytes in the NIH/NLM cell-image collection. In a subsequent study, Rajaraman et al. (11) showed that deep neural ensembles can improve malaria parasite recognition by combining complementary models. Madhu et al. (12) investigated an Inception-based capsule-network formulation for malaria parasite detection and classification, illustrating the continuing search for architectures that capture local parasite morphology more effectively. Talukdar et al. (13) combined CNN classification with Canny edge information, emphasizing the potential value of explicit structural cues around infected cells. Huq et al. (14) approached malaria-cell classification from an anomaly-detection perspective using a deep autoencoder, thereby moving beyond a purely conventional supervised classifier. Mujahid et al. (15) focused on computationally efficient deep learning for red-blood-cell smear analysis, which is particularly relevant when automated microscopy must operate under limited computational resources. Hoyos and Hoyos (16) examined the contribution of data augmentation to malaria diagnosis, reinforcing the importance of training-time variability when the available microscopic images do not fully represent field conditions.

Recent malaria-specific work has further diversified the modeling strategies. Ahamed et al. (17) developed customized interpretable CNN architectures and emphasized the need to understand the evidence used by the classifier rather than relying on accuracy alone. Singh et al. (18) combined Otsu-based segmentation with an optimized CNN, showing that foreground isolation can be integrated with learned classification. Parveen et al. (19) explicitly framed malaria diagnosis in terms of trustworthy and explainable deep learning, reflecting the increasing importance of transparent model behavior. Nakasi et al. (20) released blood-slide images collected in Uganda, providing geographically relevant data that can support evaluation beyond a single public benchmarks. Ramos-Briceño et al. (21) extended the problem from binary infection detection toward species identification, demonstrating that malaria AI must eventually address clinically meaningful heterogeneity among Plasmodium species. Shahin et al. (22) proposed a multi-model feature-fusion framework, illustrating another route for combining complementary learned representations. Ali et al. (23) introduced DANet, a lightweight dilated-attention network designed to retain discriminative information while controlling model complexity. Fu et al. (24) combined self-supervised learning with attention mechanisms, highlighting the value of representation learning when labeled microscopic data are limited. Reddy et al. (25) investigated optimized transfer learning with deep convolutional feature extractors for erythrocyte microscopy. Marques et al. (26) evaluated EfficientNet variants for malaria parasite detection, linking malaria microscopy to efficiency-oriented model scaling. Liang and Sun (27) examined adversarial robustness, showing that high nominal accuracy does not necessarily imply stable behavior under image perturbation. Collectively, these studies demonstrate that architecture, preprocessing, transfer learning, fusion, attention, and robustness can all improve malaria-image recognition; however, they address different aspects of model construction and do not eliminate the need to define an appropriate operating point for screening.

Reliability-oriented research is especially important when model outputs are intended to support clinical review. Shorten and Khoshgoftaar (28) showed why image augmentation is widely used to improve generalization by increasing training-time variability without changing the original archive. For interpretability, Grad-CAM, LIME, and SHAP provide complementary mechanisms for localizing or attributing model evidence (29–31). Guo et al. (32) demonstrated that modern neural networks can be highly discriminative while still producing poorly calibrated probabilities. Sambyal et al. (33) examined calibration specifically in medical-image classification and reinforced the need to assess probability reliability separately from classification accuracy. Gal and Ghahramani (34) provided a Bayesian interpretation of dropout that has motivated practical uncertainty-estimation strategies in deep networks. These contributions are directly relevant to the present study because explainability, uncertainty, probability calibration, and threshold selection answer different questions and should not be treated as interchangeable indicators of reliability.

Work closer to deployment has increasingly focused on image quality, computational constraints, and external validity. Zedda et al. (35) proposed an attention-based end-to-end architecture for detecting early and mature malaria parasites, emphasizing the need to preserve diagnostically relevant spatial information. Coro et al. (36) developed an open-source toolkit for thin-smear image acquisition and quality assessment, drawing attention to the fact that diagnostic AI performance begins with the quality and consistency of the acquired image. Alonso-Ramírez et al. (37) demonstrated compact malaria-cell classification on Jetson TX2 hardware, supporting the feasibility of embedded implementation. Rubio Maturana et al. (38) evaluated an AI tool together with a universal low-cost robotized microscope, bringing automated diagnosis closer to resource-constrained laboratory workflows. Martín-Ramírez et al. (39) evaluated automated microscopy in a reference laboratory, illustrating the importance of testing AI-supported workflows under operational conditions rather than only on curated image subsets. Xiong et al. (40) investigated uncertainty-guided attention for thick-smear parasite detection, integrating predictive uncertainty directly into the learning strategy. Sukumarran et al. (41) concluded in a systematic review that methodological heterogeneity and validation design remain major issues in microscopic malaria AI. Dahiya et al. (42) similarly reviewed deep-learning methods for parasite evaluation and highlighted the need for stronger clinical validation. Tiong et al. (43) emphasized diagnostic diversity, including implications for *Plasmodium knowlesi*, and cautioned against assuming that performance on one diagnostic setting transfers automatically to another. Faratisha et al. (44) synthesized diagnostic-accuracy evidence and showed the importance of interpreting pooled AI performance in light of study design and reference standards. Xing et al. (45) likewise noted in a recent scoping review that the transition from promising AI experiments to dependable malaria diagnostics requires stronger evidence of generalization and clinical integration. Recently, Owoloye et al. (46), developed a compact 13-layer CNN for field-derived thin-smear images and benchmarked it against ResNet50, VGG16, InceptionV3, and AlexNet. The authors reported 99.3% accuracy, 99.1% precision, 99.6% recall, and a 99.3% *F*_1_-score. Importantly, the study went beyond discrimination by applying temperature scaling and conformal prediction to address probability reliability and predictive uncertainty; the reported conformal analysis achieved 95.8% empirical coverage at a 95% target, with 97.6% of test cases receiving definitive single-class predictions. Plasmo3Net is therefore highly relevant to the present work, but the methodological emphasis is different. Owoloye et al. focus on calibrated probabilities and uncertainty-aware prediction sets, whereas our study focuses on how a validation-selected, sensitivity-oriented operating threshold changes false-negative burden for a compact CNN, a deeper regularized CNN, and their fixed score ensemble. These objectives are complementary: calibration concerns the reliability of predicted probabilities, whereas threshold optimization concerns the action taken from those scores.

The literature reviewed above shows that malaria AI has progressed substantially in architecture design, preprocessing, transfer learning, interpretability, robustness, calibration, uncertainty analysis, and deployment. Nevertheless, within these studies, the operating-point problem is usually secondary to the development of the classifier itself. This distinction is important for screening because two systems with similar discrimination can produce materially different numbers of missed parasitized cells when different thresholds are applied. The principal contribution of the present work is therefore not simply another CNN architecture. We provide a controlled comparison of a compact CNN, a deeper regularized CNN, and a fixed equal-weight score ensemble under both the conventional 0.50 decision rule and thresholds selected only from validation data using a recall-weighted *F*_2_ objective. We then quantify the resulting sensitivity–specificity trade-off, false-negative reduction, confidence intervals, and paired threshold-induced changes on an untouched test set. By explicitly separating discrimination, probability calibration, uncertainty, and operating-point selection, the study highlights a practical component of malaria-AI deployment that can be overlooked when performance is summarized only by accuracy or ROC–AUC. This decision-policy perspective strengthens the clinical relevance of the work while keeping the claims appropriately limited to the available image-level benchmark and the need for future patient-grouped, multicenter validation.

## 3 Materials and methods

### 3.1 Dataset and experimental protocol

#### 3.1.1 Dataset description, class percentages, and split

The complete dataset contained 27,558 segmented erythrocyte images, comprising 13,779 parasitized images (50.00%) and 13,779 uninfected images (50.00%); stratified sampling preserved this exact 50.00%–50.00% composition in the training, validation, and test subsets. Consequently, test accuracy is not inflated by a majority class in the present experiment. The balanced experimental prevalence is nevertheless a property of this benchmark rather than a representation of every clinical setting, and positive and negative predictive values must be recalculated for the local malaria prevalence before deployment.

The balanced class prior used in the experimental partition is given in Equation 1.


$$\begin{array}{l} P\left(y=1\right)=P\left(y=0\right)=\frac{13,779}{27,558}=0.50. \end{array}$$ (1)

A balanced set of 27,558 NIH/NLM thin-smear cell images was partitioned by stratified image-level sampling using a fixed random seed of 2026 in the ratio 70:15:15 for training, validation, and independent testing. Each class contributed 9,645 images to training, 2,067 to validation, and 2,067 to testing, yielding 19,290 training images (70.00%), 4,134 validation images (15.00%), and 4,134 test images (15.00%). Network weights were estimated only from training data, validation data were used for model monitoring and threshold selection, and the test set was evaluated only after all architectural, fusion, and operating-point decisions had been fixed.

Table 1 reports the exact class counts in Panel A and the permitted use of each subset in Panel B.

**Table 1:** Dataset composition, subset roles, and leakage controls.

Panel A. Stratified image-level partition				
Subset	Parasitized	Uninfected	Total	Share of dataset
Complete collection	13,779 (50.00%)	13,779 (50.00%)	27,558	100.00%
Training	9,645 (50.00%)	9,645 (50.00%)	19,290	70.00%
Validation	2,067 (50.00%)	2,067 (50.00%)	4,134	15.00%
Independent test	2,067 (50.00%)	2,067 (50.00%)	4,134	15.00%
**Panel B. Subset roles and safeguards**				
**Stage**	**Data used**	**Permitted operation**	**Locked output**	**Safeguard**
Model fitting	Training only	Gradient updates and online augmentation	CNN weights	No validation/test gradient updates
Model monitoring	Validation only	Early stopping and threshold selection	$\tau_{A}^{⋆}$, $\tau_{B}^{⋆}$, $\tau_{ens}^{⋆}$	Test labels excluded from all choices
Final assessment	Test only	One evaluation after settings were frozen	Metrics, CIs, ROC/PR curves	No retraining or threshold retuning
Independence scope	Non-overlapping image paths	File-level separation across subsets	Split manifests; seed 2026	Patient/slide independence not claimed

The partition roles were fixed before model fitting. The training subset was the only source of gradient updates. The validation subset supported early stopping, threshold optimization, and any model-selection decision. The test subset was reserved for the final evaluation after the architectures, score-fusion rule, threshold grid, and tie-breaking rule had been fixed. This separation limits direct test-set leakage, although it does not substitute for patient-grouped external validation.

#### 3.1.2 Unit of analysis and independence

The unit of analysis in the publicly distributed NIH/NLM release is the segmented cell image; therefore, the manifests guarantee that the same image file does not occur in more than one subset, but they do not establish patient-level or slide-level independence because a reliable grouping identifier is unavailable. Cells originating from the same patient or specimen may consequently share acquisition characteristics across subsets, potentially producing a more optimistic estimate than a patient-disjoint evaluation; patient- and site-grouped external validation is therefore required before clinical generalization.

#### 3.1.3 Visual inspection of the data

**Figure 1:**
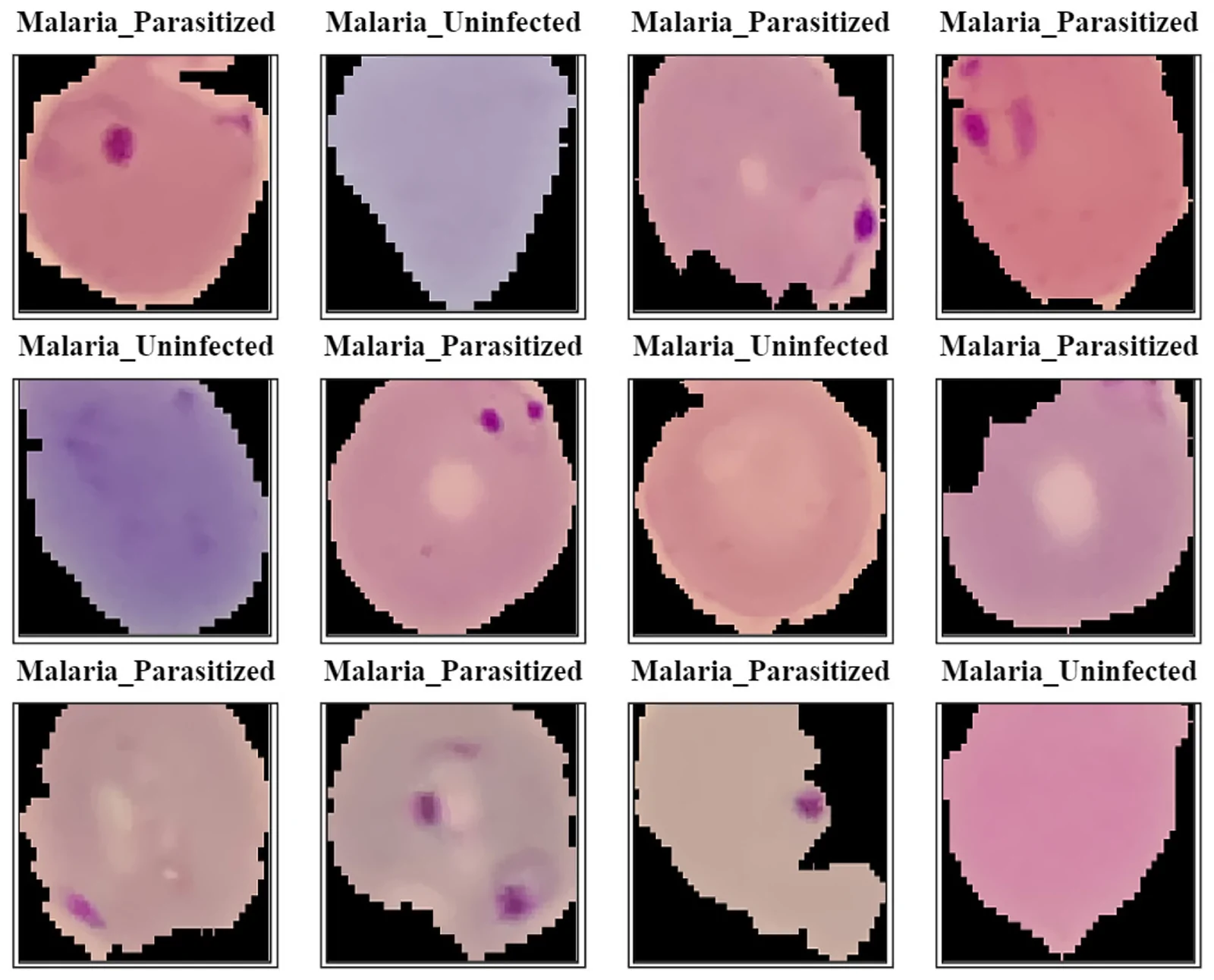
Representative raw malaria microscopy images. The mosaic illustrates the natural variability of cell size, stain intensity, texture, and parasite appearance across the dataset.

Figure 1 presents a representative mosaic of the malaria cell images. The visual diversity of stain intensity, cell boundary quality, cytoplasmic color, and parasite-like chromatic texture shows why a robust classifier must tolerate significant microscopic variation. The balanced class counts described above confirm that both classes are equally represented, making the comparison between sensitivity and specificity meaningful.

This visual diversity reinforces the need for a model that is not only accurate on average, but also sensitive to subtle parasite-associated structures under variable staining and cell-boundary conditions.

### 3.2 Proposed methodology

#### 3.2.1 CNN input preparation, resizing rationale, and augmentation

**Figure 2:**
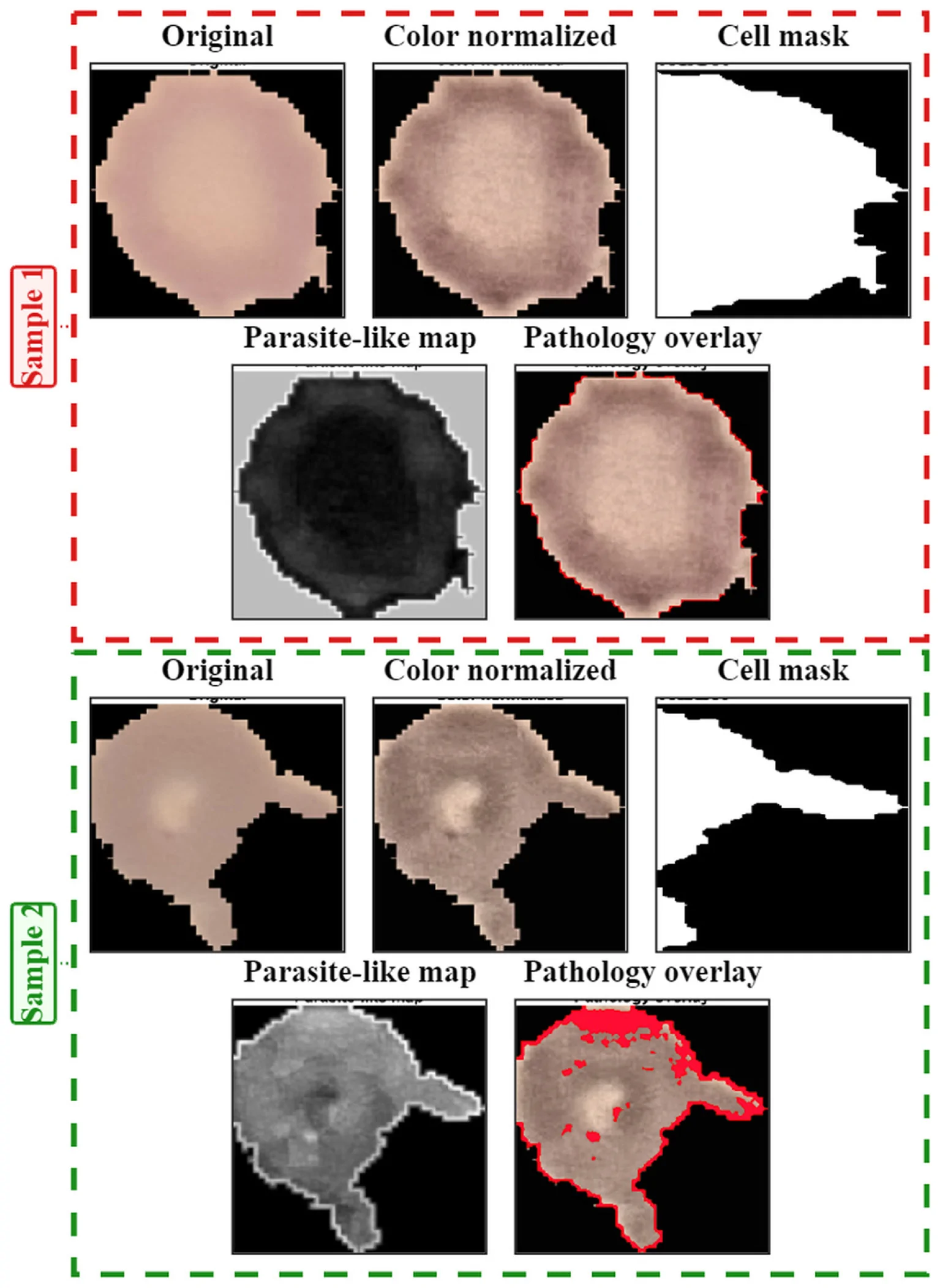
Image-treatment pipeline. The transformation from raw image to color-normalized image, cell mask, parasite-like map, and pathology overlay provides a clear visual representation of suspected parasite-related structures.

**Figure 3:**
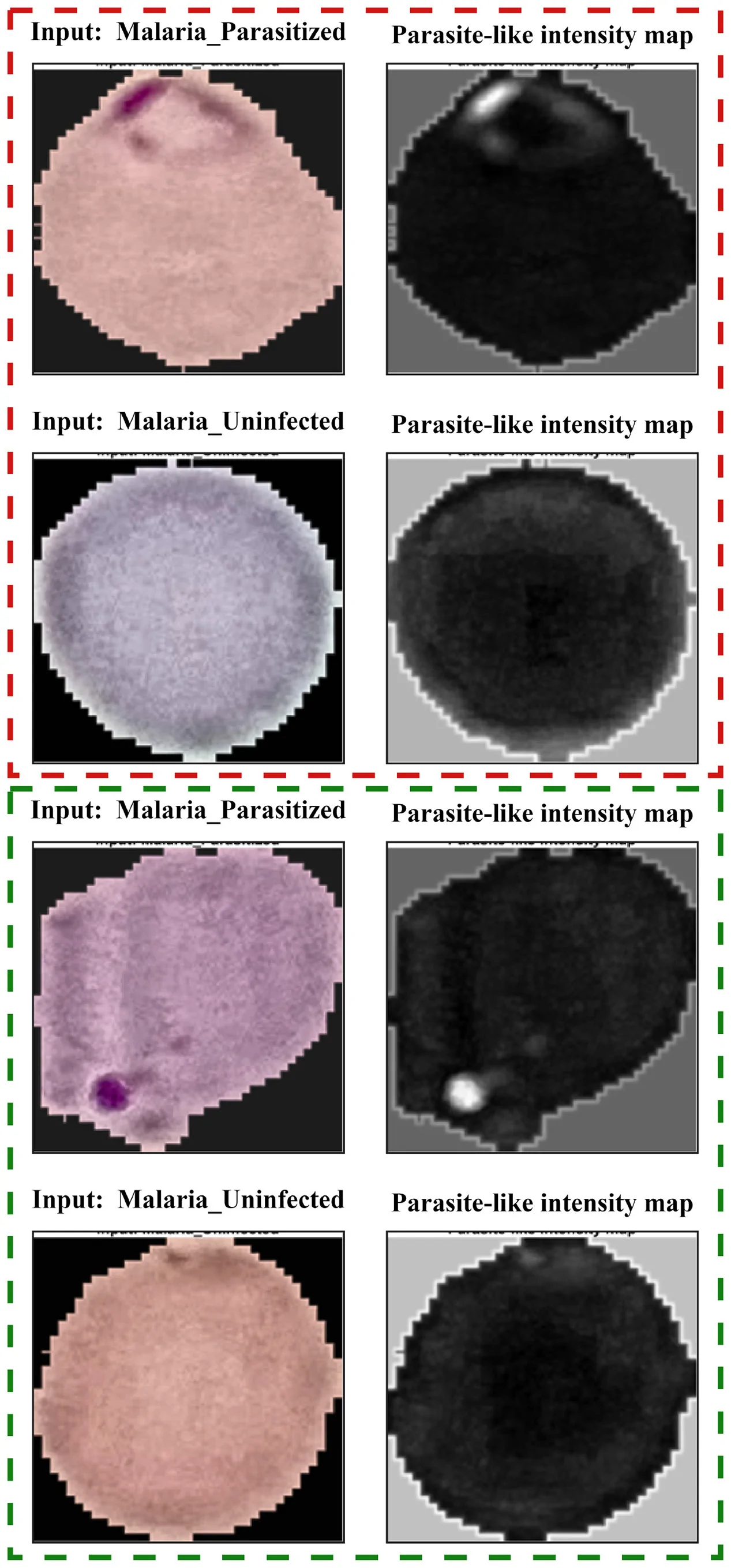
Detailed parasite-like texture maps. The figure shows that the proposed image-treatment module emphasizes local color and texture irregularities that may correspond to parasite-containing structures.

The actual CNN input path consists of image decoding, conversion of grayscale inputs to three channels when necessary, resizing to 224 × 224 × 3, zero-center normalization in the input layer, and training-only online augmentation. The stain-normalized masks and parasite-like maps shown in Figures 2, 3 are visualization aids and were not supplied as additional training channels. This distinction prevents the image-treatment illustration from being mistaken for an undocumented learned preprocessing stage.

All decoded images were resized deterministically to 224 × 224 × 3 because both CNNs require a fixed spatial tensor for mini-batch optimization and an identical resolution for fair score-level comparison. This resolution preserves the visible erythrocyte boundary, chromatin-like inclusions, and parasite-related texture while substantially reducing memory use and computation relative to heterogeneous native dimensions; the same operation was applied to training, validation, and test images without using labels or test outcomes. Interpolation can modify extremely fine texture; consequently, verification at native acquisition resolution and on external-site images remains an important future robustness analysis. The identical resize applied to both methods nevertheless preserves the internal fairness of the present comparison.

Data augmentation was generated online and applied exclusively to the training subset, so it did not alter the original size or composition of the NIH/NLM archive. Method A used random rotations in [−14°, 14°], horizontal and vertical reflections, translations in [−8, 8] pixels along both axes, and independent scaling in [0.92, 1.08], whereas Method B used the stronger ranges [−22°, 22°], [−12, 12] pixels, and [0.88, 1.12] with the same reflections. These transformations represent plausible variations in cell orientation, centering, and apparent size because isolated erythrocytes have no fixed anatomical up direction; no augmentation, class-conditional synthesis, artificial parasite insertion, stain-changing transformation, or test-time augmentation was applied to validation or test images.

#### 3.2.2 Visualization-only stain-aware image treatment

For qualitative inspection, an RGB image is resized to 256 × 256, transformed to the CIELAB color space, and locally contrast-normalized in the luminance channel. A cell-support mask suppresses the background, while a parasite-emphasis response combines saturation, darkness, chromatic deviation, local texture, and gradient magnitude. For an image channel *c*, standardized intensity can be written as


$$\begin{array}{c} {\tilde{I}}_{c}\left(u,v\right)=\frac{I_{c}\left(u,v\right)-\mu_{c}}{\sigma_{c}+\varepsilon }, \end{array}$$ (2)

$$\begin{array}{c} R\left(u,v\right)=M\left(u,v\right)\Phi \left(\tilde{\text{I}}\left(u,v\right),\nabla \tilde{\text{I}}\left(u,v\right)\right). \end{array}$$ (3)

The channel standardization and parasite-emphasis response are defined in Equations 2 and 3, respectively.

These maps highlight suspicious regions for qualitative review; they do not replace the learned CNN features and do not alter the original dataset size. Figure 2 shows the successive visual-processing steps, and Figure 3 presents representative local response maps; neither figure supplies additional input channels to the CNNs.

#### 3.2.3 Method A, Method B, and score-level ensemble

The two CNNs were designed as complementary models rather than as minor variants of the same network. Method A serves as a lower-capacity baseline intended to learn progressively more abstract erythrocyte features with a short four-stage hierarchy. Method B increases depth and regularization so that multiple convolutions can refine local color–texture patterns before each spatial down-sampling step. Both models receive exactly the same 224 × 224 × 3 image tensor and differ only in architecture, augmentation strength, and the pre-specified training settings reported in Table 2.

**Table 2:** CNN architecture, training, augmentation, and decision settings.

Panel A. Architecture		
Component	Method A	Method B
Input	224 × 224 × 3; zero-center normalization	224 × 224 × 3; zero-center normalization
Convolutions	32–64–128–256 filters	32–32–64–64–128–128–256–256–384 filters
Blocks	3 × 3 convolution, BN, ReLU; max pooling after stages 1–3	Paired 3 × 3 convolution, BN, ReLU; max pooling after blocks 1–4
Classifier	GAP; dropout 0.30/0.40; two-class softmax	GAP; dropout 0.20/0.30/0.50; two-class softmax
Trainable parameters	Approximately 0.390 million	Approximately 2.061 million
**Panel B. Training, augmentation, and decision rules**		
**Setting**	**Method A**	**Method B**
Optimizer and epochs	Adam; batch 64; 14 epochs; LR 3 × 10^−4^	Adam; batch 64; 18 epochs; LR 2 × 10^−4^
Regularization	$L_{2}=10^{-4}$; gradient limit 5; patience 5	$L_{2}=2\times 10^{-4}$; gradient limit 5; patience 5
LR schedule	Factor 0.30 every 5 epochs	Factor 0.30 every 6 epochs
Online augmentation	Rotation ±14°; translation ±8 px; scale 0.92–1.08; H/V reflection	Rotation ±22°; translation ±12 px; scale 0.88–1.12; H/V reflection
Evaluation and fusion	Batch 128; canonical class order; *S*_*ens*_ = 0.5*S*_*A*_ + 0.5*S*_*B*_; threshold grid 0.05:0.005:0.95; validation-only *F*_2_ maximization	
Tie rule	Highest *F*_2_, then highest sensitivity, then highest threshold; all settings fixed before test evaluation	

Method A contains four convolutional stages with 32, 64, 128, and 256 filters, respectively. Every convolution uses a 3 × 3 kernel with same padding, followed by batch normalization and a rectified linear unit (ReLU). A 2 × 2 max-pooling layer with stride two follows the first three stages, reducing the feature-map size from 224 × 224 to 112 × 112, 56 × 56, and 28 × 28 while increasing the channel depth. The 256-filter stage operates at the final spatial scale. Dropout with probability 0.30 is applied before global average pooling, followed by dropout of 0.40, a two-unit fully connected layer, softmax, and the binary classification output. This architecture contains approximately 0.390 million trainable parameters. The compact design provides a deliberately restrained reference model with fewer parameters and less opportunity to fit dataset-specific detail.

Method B is a deeper nine-convolution architecture organized into four paired convolutional blocks followed by a final 384-filter convolution. The paired blocks use 32–32, 64–64, 128–128, and 256–256 filters. Each convolution again uses a 3 × 3 kernel, same padding, batch normalization, and ReLU; max pooling is applied after each pair. Consequently, two non-linear transformations are learned at a given spatial scale before resolution is reduced, so that Method B can refine local parasite-associated texture and chromatic patterns at greater depth than Method A. Dropout is introduced progressively after the 128-filter block (0.20), after the 256-filter block (0.30), and before the final classifier (0.50). The 384-filter layer is followed by global average pooling, a two-unit fully connected layer, softmax, and the binary output. The resulting model has approximately 2.061 million trainable parameters. The additional depth is therefore accompanied by stronger regularization rather than being used solely to increase capacity.

For both networks, the softmax component corresponding to the parasitized class is treated as a continuous score, denoted *S*_*A*_ for Method A and *S*_*B*_ for Method B. The ensemble does not introduce an additional learned classifier: its score is fixed *a priori* as *S*_*ens*_ = 0.5*S*_*A*_ + 0.5*S*_*B*_. Equal weighting was chosen to avoid fitting another set of parameters to the validation or test data. Thus, the comparison isolates three decision sources–the compact model, the deeper regularized model, and a simple score fusion–while keeping the final operating-point procedure identical across them.

Training settings were also fixed separately for the two capacity profiles. Method A used Adam with an initial learning rate of 3 × 10^−4^ for at most 14 epochs, whereas Method B used 2 × 10^−4^ for at most 18 epochs. Both used a training mini-batch size of 64, evaluation batch size of 128, validation-based early stopping with patience five, gradient clipping at five, batch normalization after every convolution, and piecewise learning-rate reduction by a factor of 0.30. The *L*_2_ coefficients were 10^−4^ for Method A and 2 × 10^−4^ for Method B. Method B also received the stronger online augmentation described above. These differences were specified before final test evaluation and are intended to match model capacity with regularization rather than to tune on the test set. Table 2 gives the complete layer sequence and hyperparameters in a compact reproducibility format.

**Figure 4:**
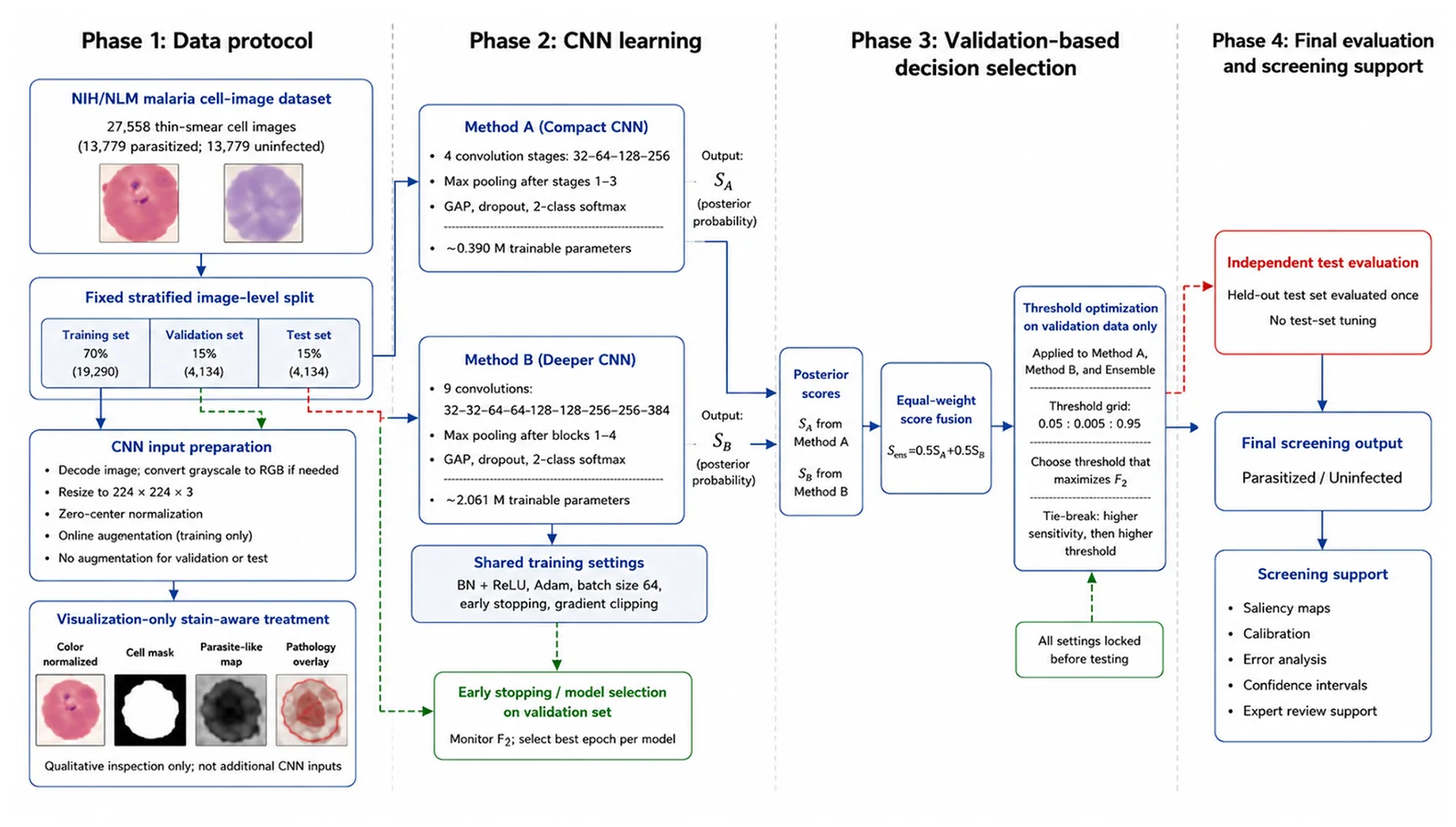
Workflow of the proposed malaria-screening framework.

The computational workflow shown in Figure 4 begins with the fixed 70:15:15 stratified split, followed by deterministic resizing and normalization and by online augmentation applied only to training images. Method A and Method B independently generate parasitized-class probabilities, which are combined through the fixed rule *S*_*ens*_ = 0.5*S*_*A*_ + 0.5*S*_*B*_; each operating threshold is then selected exclusively from validation data by maximizing *F*_2_. All settings are locked before the independent test set is evaluated once. The parasite-like maps and saliency outputs in the four-phase figure are supporting visual checks and are not additional CNN input channels; Tables 1, 2 provide the corresponding split, architecture, training, augmentation, and leakage-control details.

#### 3.2.4 Sensitivity-oriented threshold optimization

False negatives were prioritized for screening. Each model was evaluated at the conventional threshold of 0.5 and at a threshold selected exclusively on the validation set from *T* = {0.05, 0.055, …, 0.95} by maximizing the recall-weighted *F*_2_-score:


$$\begin{array}{c} F_{2}=\frac{5\text{ Precision Sensitivity}}{4\text{ Precision}+\text{Sensitivity}}, \end{array}$$ (4)

$$\begin{array}{c} \tau^{⋆}=\underset{\tau \in T}{arg max}F_{2}\left(\tau D_{\text{val}}\right). \end{array}$$ (5)

The recall-weighted F2 objective and validation-only threshold-selection rule are defined in Equations 4 and 5, respectively.

Ties were resolved by higher validation sensitivity and then by the highest remaining threshold. Test labels were not used for threshold selection.

#### 3.2.5 Probability calibration and distinction from threshold optimization

Probability calibration and threshold selection are distinct. Calibration asks whether predicted probabilities correspond to observed event frequencies, whereas threshold selection converts a score into a class decision. Maximizing *F*_2_ changes only the operating point; no post-hoc calibration transformation was fitted, so the reported thresholds operate on the native softmax scores.

For external use, any recalibration (e.g., temperature scaling) should be fitted only on a separate local calibration cohort, followed by task-specific threshold selection before evaluation on an untouched test cohort. The Plasmo3Net study illustrates the complementary use of temperature scaling and conformal prediction for malaria microscopy (46); these procedures are not components of the present experiments.

### 3.3 Evaluation metrics and statistical analysis

Accuracy, sensitivity, specificity, precision, negative predictive value (NPV), false-negative rate (FNR), Matthews correlation coefficient (MCC), balanced accuracy, *F*_1_, *F*_2_, receiver-operating-characteristic area under the curve (ROC–AUC), and precision–recall area under the curve (PR–AUC) were computed. All abbreviations are defined at first use and are also listed in the nomenclature table.

The classification metrics used in this analysis are defined in Equations 6-8.


$$\text{Accuracy}=\frac{TP+TN}{TP+TN+FP+FN},\text{ Sensitivity}=\frac{TP}{TP+FN},$$ (6)

$$\text{Specificity}=\frac{TN}{TN+FP},\text{ Precision}=\frac{TP}{TP+FP},$$ (7)

$$\text{FNR}=\frac{FN}{TP+FN},\text{ NPV}=\frac{TN}{TN+FN}.$$ (8)

Two-sided 95% Wilson score confidence intervals were calculated for binomial proportions, including accuracy, sensitivity, specificity, precision, NPV, and FNR. For an observed proportion $\hat{p}=k/n$ and *z* = 1.96, the Wilson interval is

The Wilson score interval used for binomial proportions is given in Equation 9.


$$\begin{array}{c} \frac{\hat{p}+z^{2}/\left(2n\right)\;\pm \;z\sqrt{\hat{p}\left(1-\hat{p}\right)/n+z^{2}/\left(4n^{2}\right)}}{1+z^{2}/n}. \end{array}$$ (6)

ROC–AUC confidence intervals were estimated with the Hanley–McNeil large-sample approximation. Let *A* denote AUC, *n*_1_ the number of parasitized images, *n*_0_ the number of uninfected images, *Q*_1_ = *A*/(2 − *A*), and $Q_{2}=2A^{2}/\left(1+A\right)$. The standard error is

The ROC-AUC standard-error expression is given in Equation 10.


$$\begin{array}{c} \text{SE}\left(A\right)=\sqrt{\frac{A\left(1-A\right)+\left(n_{1}-1\right)\left(Q_{1}-A^{2}\right)+\left(n_{0}-1\right)\left(Q_{2}-A^{2}\right)}{n_{1}n_{0}}}, \end{array}$$ (7)

and the reported interval is *A* ± 1.96SE(*A*), truncated to [0, 1].

Default and optimized decisions for the same network are derived from the same scores. Their sensitivity change was therefore evaluated with an exact paired McNemar/binomial test on the parasitized subset. Lowering the threshold produced nested positive predictions: Method A corrected 58 previously missed parasitized cells, Method B corrected 116, and the ensemble corrected 96, with no reverse changes among positive cases. The exact two-sided *p*-values were 6.94 × 10^−18^, 2.41 × 10^−35^, and 2.52 × 10^−29^, respectively; all remained significant after Holm adjustment for three comparisons. The corresponding absolute sensitivity gains were 2.81, 5.61, and 4.64 percentage points, and the relative reductions in false negatives were 40.28%, 65.54%, and 60.00%.

Direct pairwise inferential comparison among Method A, Method B, and the ensemble was not computed because the retained aggregate outputs do not contain the aligned per-image predictions and score vectors required to construct discordant pairs for McNemar testing or the paired covariance structure required for a DeLong ROC–AUC comparison. Such *p*-values cannot be recovered validly from marginal confusion matrices or AUC values alone. Between-model rankings are therefore interpreted descriptively with 95% confidence intervals, and no claim of statistical superiority is made. The supplementary statistical-audit implementation accepts aligned per-image scores and performs paired comparisons when those outputs are available.

## 4 Results

### 4.1 Training behavior

**Figure 5:**
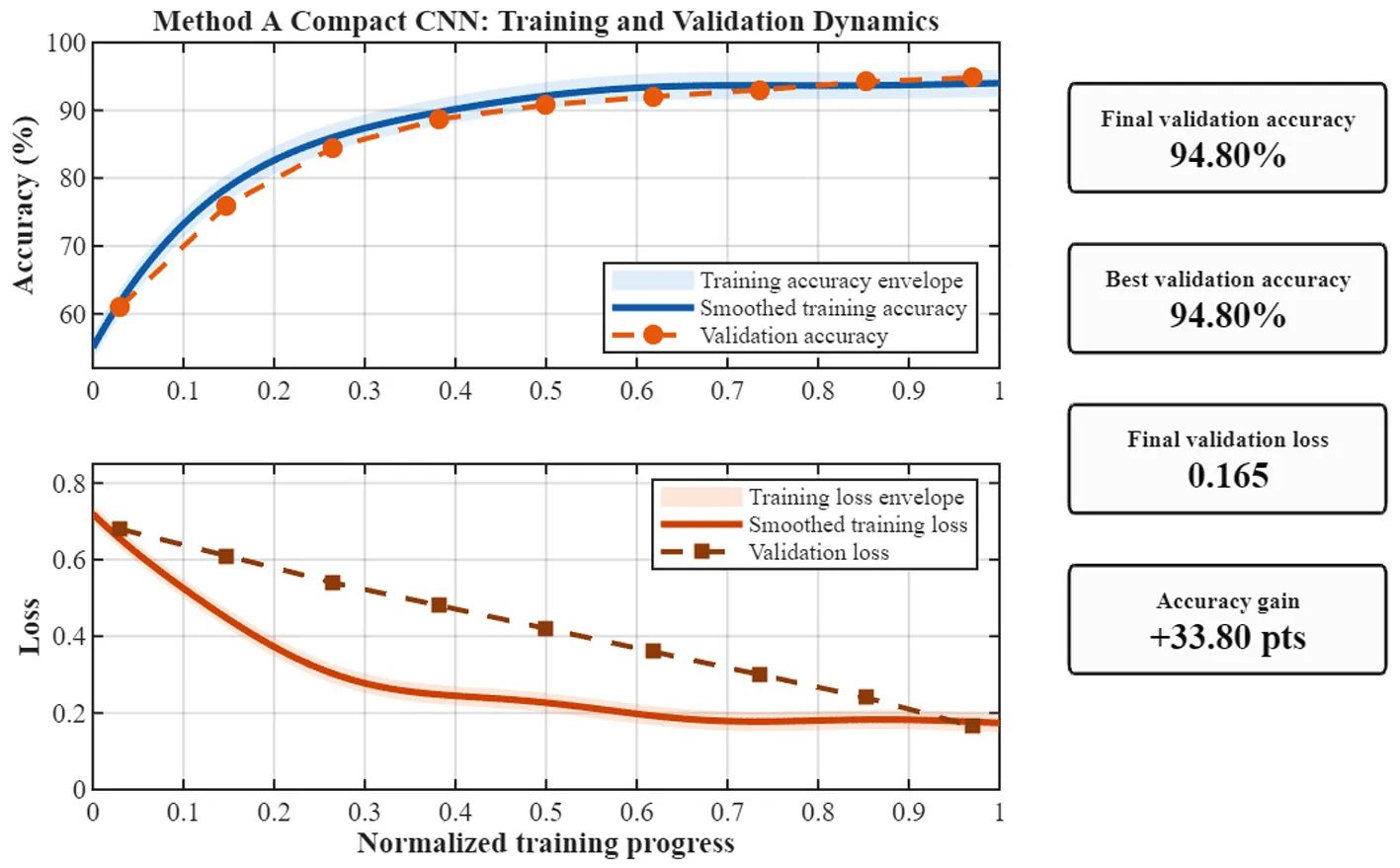
Training and validation behavior of Method A, showing the convergence of the compact CNN across the recorded optimization trajectory.

**Figure 6:**
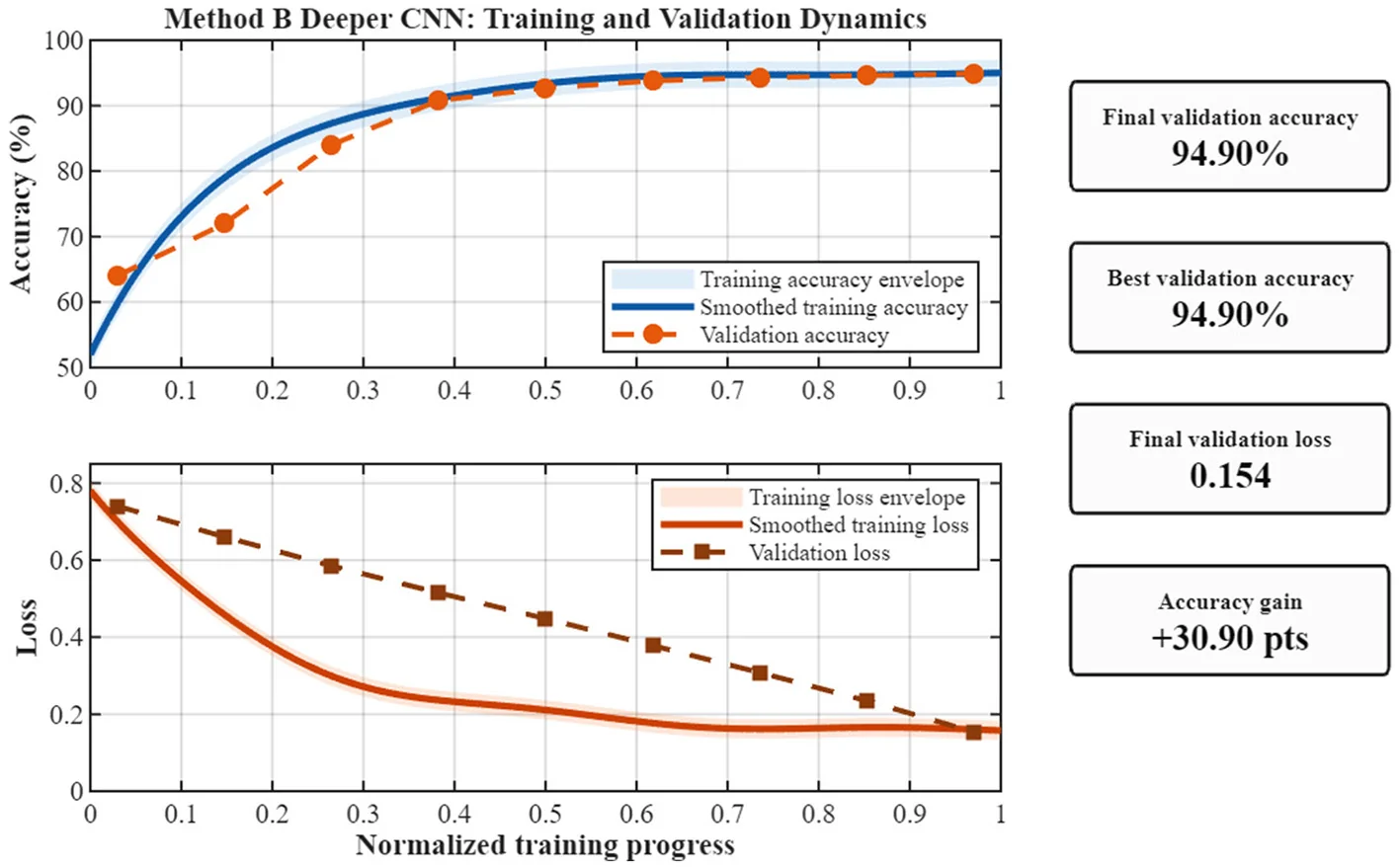
Training and validation behavior of Method B, showing stable convergence of the deeper CNN and its terminal validation performance.

Both networks reached stable learning regimes, but their terminal behavior reveals an important distinction between point accuracy and probability ranking. Method A converged rapidly and produced a strong conventional classifier, as reflected by the compact training trajectory in Figure 5. Method B exhibited a similarly stable optimization pattern (Figure 6) while generating better ordered posterior scores. This stronger ranking quality, rather than default-threshold accuracy alone, explains its later advantage in ROC–AUC, PR–AUC, and sensitivity-oriented threshold selection.

### 4.2 Screening error-decomposition analysis

**Figure 7:**
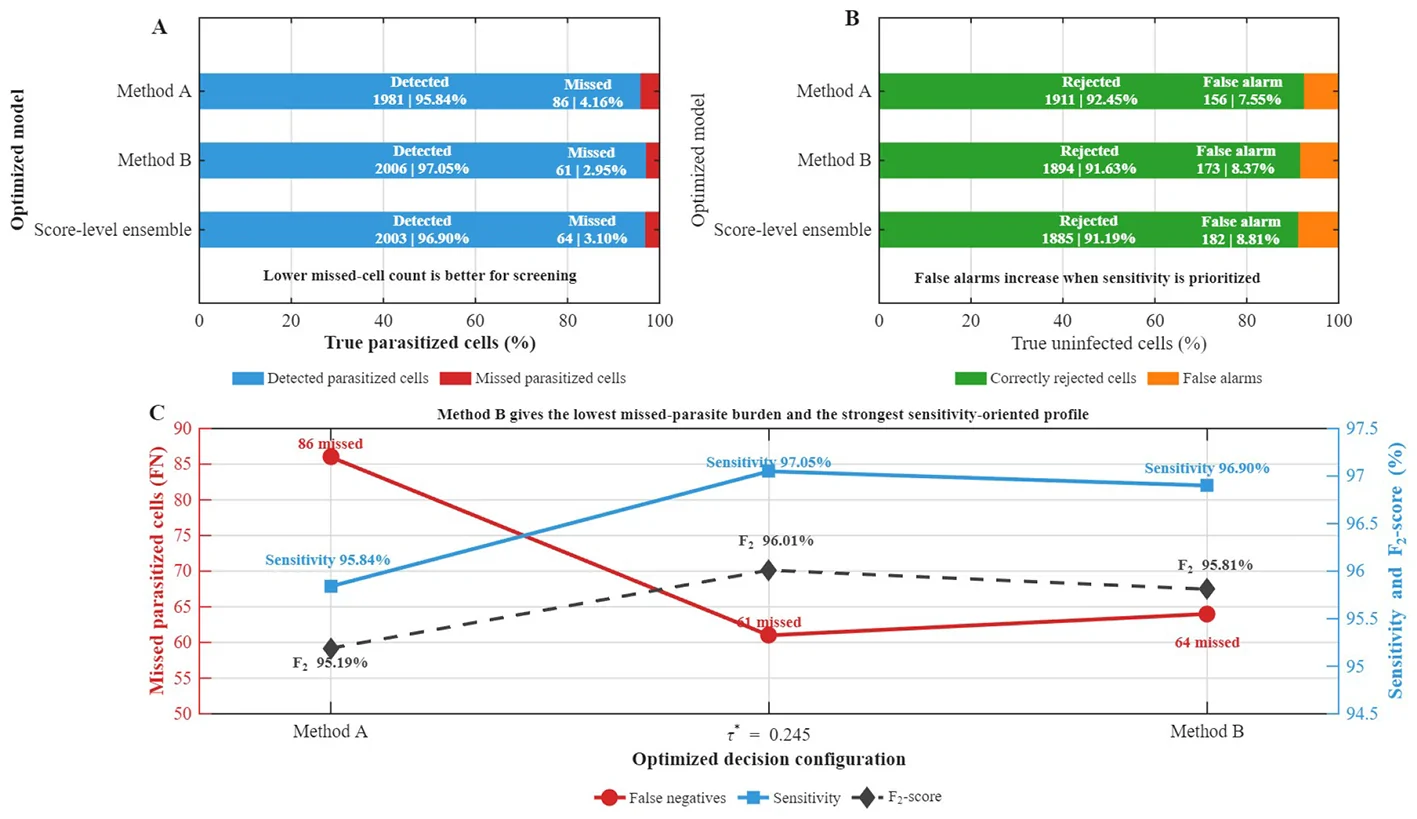
Integrated screening-error dashboard for the optimized decision thresholds. The upper panels decompose parasitized cells into detected and missed outcomes and uninfected cells into correctly rejected and false-alarm outcomes. The lower panel summarizes the screening trade-off through false-negative count, sensitivity, and *F*_2_-score. Method B at τ^⋆^ = 0.050 detects 2,006 of 2,067 parasitized cells and misses only 61, giving the lowest false-negative burden, the highest sensitivity (97.05%), and the highest *F*_2_-score (96.01%) among the optimized configurations. **(A)** parasitized outcomes; **(B)** uninfected outcomes; **(C)** FN/sensitivity/F2 trade-off.

The optimized operating points expose the practical consequence of threshold selection more clearly when the error counts are viewed as screening outcomes rather than as three separate screening error-decomposition summaries. Figure 7 therefore summarizes the optimized decisions of Method A, Method B, and the score-level ensemble in a single integrated dashboard. Among the 2,067 parasitized test cells, Method A detects 1,981 and misses 86, Method B detects 2,006 and misses only 61, and the ensemble detects 2,003 and misses 64. This means that the optimized Method B threshold reduces the missed-parasite burden by 25 cells relative to optimized Method A and by three cells relative to the optimized ensemble. In screening terms, this is the most important result of the experiment, because an additional false-positive review is generally less harmful than an overlooked parasitized cell.

The same figure also shows the price paid for this safer operating point. Method B produces 173 false alarms among the uninfected cells, compared with 156 for Method A and 182 for the ensemble. This trade-off is expected after sensitivity-oriented threshold migration: more cells are flagged for review, but fewer infected cells are missed. The central finding is therefore not that Method B dominates every metric, but that it provides the strongest screening compromise, with 97.05% sensitivity and a 96.01% *F*_2_-score at τ^⋆^ = 0.050. The ensemble remains very close, reaching 96.90% sensitivity and a 95.81% *F*_2_-score, but it does not surpass the optimized deeper CNN. Thus, the single dashboard in Figure 7 makes the main clinical message explicit: validation-based operating-point design can be more decisive than adding a fusion stage when the main objective is to reduce missed parasitized cells.

### 4.3 Ranking quality and accuracy-oriented comparison

**Figure 8:**
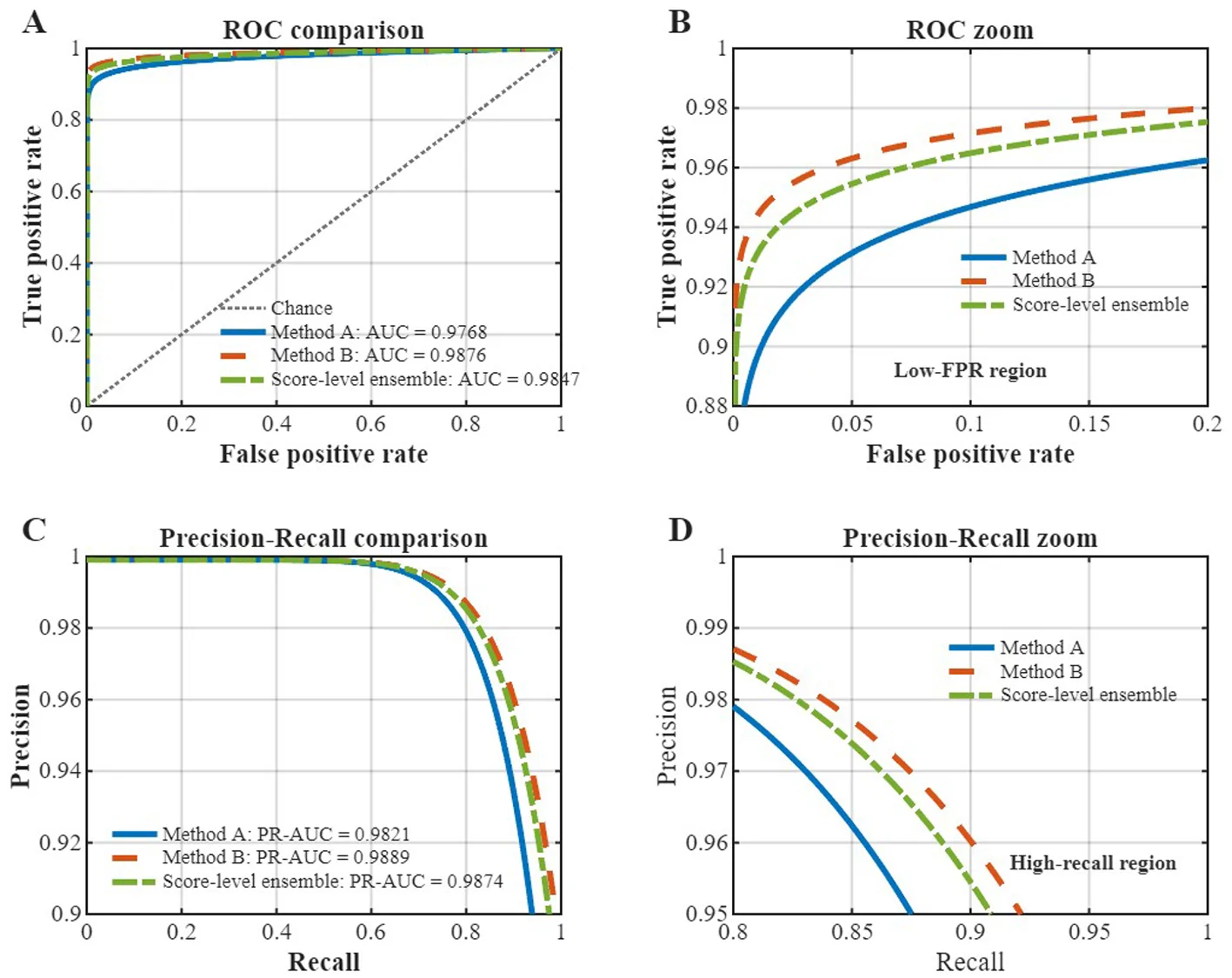
Unified ROC and precision–recall evidence with zoomed operating regions. Method B achieves the strongest threshold-independent ranking quality, with ROC–AUC of 0.9876 and PR–AUC of 0.9889. The ensemble remains close but lower (ROC–AUC 0.9847; PR–AUC 0.9874), whereas Method A obtains ROC–AUC 0.9768 and PR–AUC 0.9821. The zoomed panels show that Method B maintains the most favorable separation in the low-FPR and high-recall regions, supporting its selection as the final sensitivity-oriented screening model. **(A)** ROC; **(B)** ROC zoom; **(C)** precision-recall; **(D)** precision-recall zoom.

Threshold-independent ranking analysis confirms that the deeper architecture produces the most discriminative score distribution. Figure 8 combines the ROC and precision–recall evidence with zoomed regions so that the global ranking and the clinically relevant high-sensitivity behavior can be assessed together. Method B attains the highest ROC–AUC (0.9876) and PR–AUC (0.9889), followed by the ensemble (ROC–AUC 0.9847; PR–AUC 0.9874) and Method A (ROC–AUC 0.9768; PR–AUC 0.9821). The zoomed ROC panel emphasizes the low-false-positive-rate region, while the zoomed precision–recall panel emphasizes the high-recall region where screening models are usually expected to operate. This combined view explains why Method B can be shifted toward a more aggressive sensitivity-oriented threshold with less loss of practical screening quality than the alternatives.

Ahamed et al. (17) strengthened customized CNNs with visual explanation tools. Parveen et al. (19) added trustworthy reporting and independent-dataset analysis. Mujahid et al. (15) concentrated on an efficient architecture. Shahin et al. (22) pursued feature fusion and voting across several models. Ali et al. (23) reduced computational complexity through a lightweight dilated-attention network. These directions establish the value of architecture and representation learning; Figure 8 adds a different result: once a strong score distribution is available, the decision threshold determines whether the system behaves as an accuracy-oriented classifier or as a sensitivity-oriented screening assistant.

### 4.4 Final quantitative comparison

**Figure 9:**
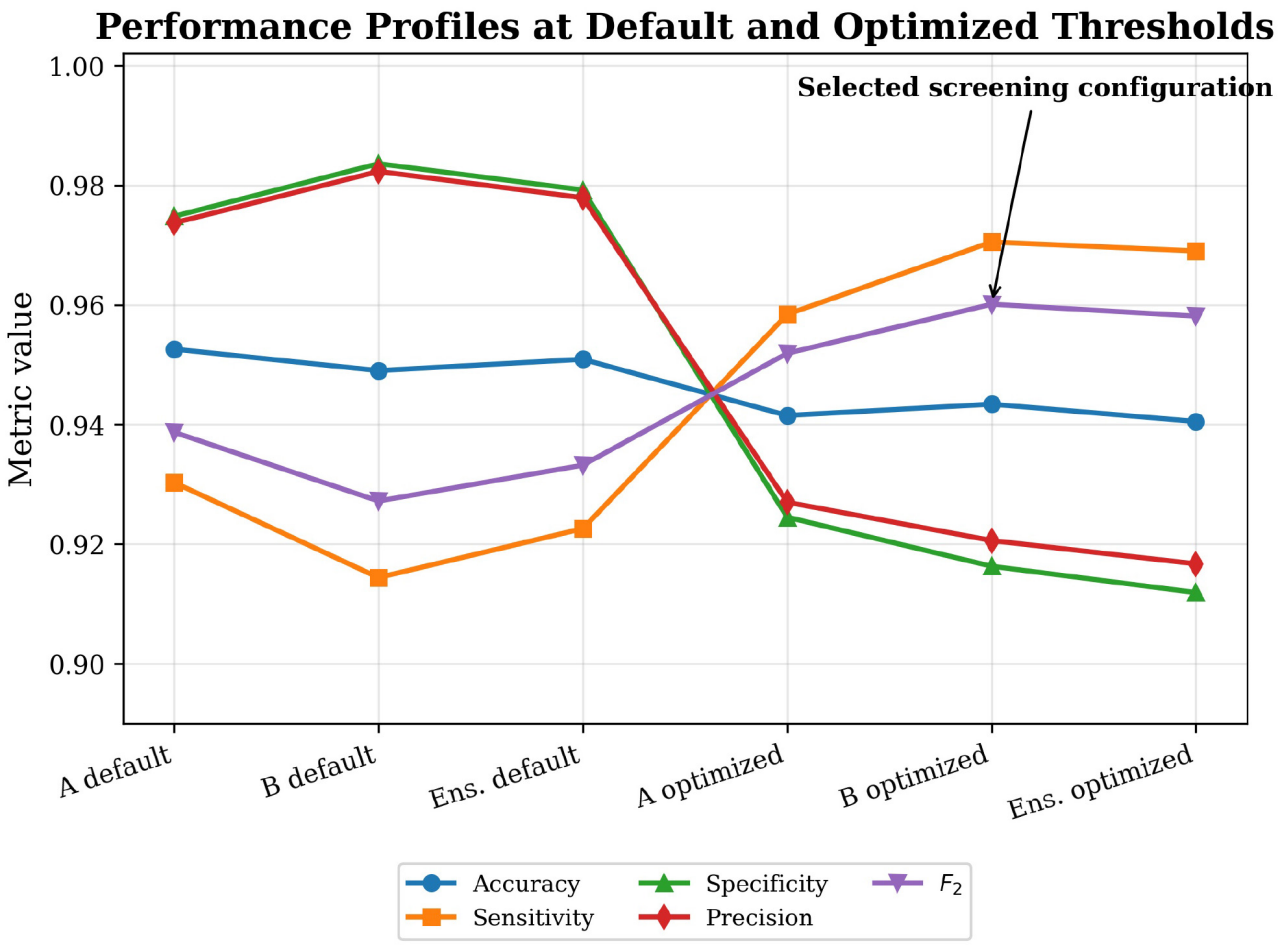
Comparative performance profile across the principal screening metrics. Method A at the default threshold has the highest accuracy-oriented values, whereas optimized Method B has the highest screening-oriented point estimates. Direct between-model statistical superiority is not claimed.

The six model–decision configurations show an objective-dependent descriptive ranking. At τ = 0.5, Method A has the highest accuracy (95.26%) and specificity (97.48%). After validation-only threshold selection, Method B has the highest sensitivity (97.05%), *F*_2_ (96.01%), ROC–AUC (0.9876), and PR–AUC (0.9889), with the fewest false negatives (61). Table 3 and Figure 9 summarize these trade-offs. These between-model differences are treated as point-estimate comparisons rather than statistically established superiority because aligned per-image outputs were not available for direct paired testing.

Table 3Independent-test performance and statistical evidence for threshold selection.

**Table:** 

Panel A. Performance of all model–decision configurations									
Configuration	τ	Accuracy (95% CI)	Sensitivity (95% CI)	Specificity (95% CI)	Precision (95% CI)	*F*_2_	FN	ROC–AUC (95% CI)	PR–AUC
Method A default	0.500	0.9526 (0.9457–0.9587)	0.9303 (0.9185–0.9405)	0.9748 (0.9672–0.9808)	0.9737 (0.9656–0.9799)	0.9387	144	0.9768 (0.9721–0.9815)	0.9821
Method B default	0.500	0.9490 (0.9418–0.9553)	0.9144 (0.9015–0.9257)	0.9836 (0.9771–0.9882)	0.9823 (0.9754–0.9873)	0.9272	177	0.9876 (0.9842–0.9910)	0.9889
Ensemble default	0.500	0.9509 (0.9439–0.9571)	0.9226 (0.9103–0.9333)	0.9792 (0.9721–0.9845)	0.9779 (0.9704–0.9836)	0.9332	160	0.9847 (0.9809–0.9885)	0.9874
Method A optimized	0.245	0.9415 (0.9339–0.9482)	0.9584 (0.9489–0.9662)	0.9245 (0.9123–0.9351)	0.9270 (0.9152–0.9373)	0.9519	86	0.9768 (0.9721–0.9815)	0.9821
**Method B optimized**	**0.050**	0.9434 (0.9359–0.9500)	**0.9705 (0.9623–0.9770)**	0.9163 (0.9036–0.9275)	0.9206 (0.9085–0.9312)	**0.9601**	**61**	**0.9876 (0.9842–0.9910)**	**0.9889**
Ensemble optimized	0.135	0.9405 (0.9329–0.9473)	0.9690 (0.9607–0.9757)	0.9119 (0.8990–0.9234)	0.9167 (0.9044–0.9276)	0.9581	64	0.9847 (0.9809–0.9885)	0.9874

**Table:** 

Panel B. Screening-centered effect of validation-selected thresholds							
Configuration	NPV (95% CI)	FNR (95% CI)	Sensitivity gain	FN reduction	Exact paired *p*	Holm-adjusted *p*	MCC
Method A optimized	0.9569 (0.9471–0.9650)	0.0416 (0.0338–0.0511)	+2.81 points	144 → 86 (40.28%)	6.94 × 10^−18^	6.94 × 10^−18^	0.8834
**Method B optimized**	**0.9688 (0.9601–0.9756)**	**0.0295 (0.0230–0.0377)**	**+5.61 points**	**177 → 61 (65.54%)**	2.41 × 10^−35^	7.23 × 10^−35^	0.8881
Ensemble optimized	0.9672 (0.9583–0.9742)	0.0310 (0.0243–0.0393)	+4.64 points	160 → 64 (60.00%)	2.52 × 10^−29^	5.04 × 10^−29^	0.8824

FN reduction and paired *p*-values compare each optimized threshold with the same model at τ = 0.5. They quantify the threshold effect within each model and do not constitute pairwise tests between different architectures. Bold values indicate the selected Method B point estimates; they do not imply statistical superiority.

Tables 1, 2 contain fixed design quantities, whereas Table 3 reports two-sided 95% confidence intervals for estimable performance measures, using Wilson intervals for accuracy, sensitivity, specificity, precision, NPV, and FNR and the Hanley–McNeil approximation for ROC–AUC. For the selected Method B operating point, the estimates are sensitivity 0.9705 (95% CI: 0.9623–0.9770), specificity 0.9163 (0.9036–0.9275), precision 0.9206 (0.9085–0.9312), NPV 0.9688 (0.9601–0.9756), FNR 0.0295 (0.0230–0.0377), and ROC–AUC 0.9876 (0.9842–0.9910), so the reported performance is presented together with its sampling uncertainty.

On the independent test set, Method A at τ^⋆^ = 0.245 achieved 95.84% sensitivity (95% CI: 94.89%–96.62%), 92.45% specificity (91.23%–93.51%), *F*_2_ = 95.19%, and 86 false negatives; relative to τ = 0.5, this corresponds to a 2.81-percentage-point sensitivity gain, a reduction from 144 to 86 missed parasitized cells (40.28%), and an exact paired *p* = 6.94 × 10^−18^. Method B at τ^⋆^ = 0.050 achieved 94.34% accuracy (93.59%–95.00%), 97.05% sensitivity (96.23–%97.70%), 91.63% specificity (90.36%–92.75%), 92.06% precision (90.85%–93.12%), 96.88% NPV (96.01%–97.56%), *F*_2_ = 96.01%, ROC–AUC 0.9876 (0.9842–0.9910), PR–AUC 0.9889, and 61 false negatives; compared with the same network at τ = 0.5, sensitivity increased by 5.61 percentage points and false negatives decreased from 177 to 61 (65.54%), with *p* = 2.41 × 10^−35^. The ensemble at τ^⋆^ = 0.135 achieved 96.90% sensitivity (96.07%–97.57%), 91.19% specificity (89.90%–92.34%), *F*_2_ = 95.81%, ROC–AUC 0.9847 (0.9809–0.9885), and 64 false negatives, representing a 4.64-percentage-point sensitivity gain and a reduction from 160 to 64 false negatives (60.00%), with *p* = 2.52 × 10^−29^. All three effects remained significant after Holm correction. Method B has the highest screening-oriented point estimates, but its sensitivity interval overlaps that of the ensemble and the false-negative counts differ by only three images. This numerical difference is therefore not interpreted as statistically established superiority.

### 4.5 Threshold-optimization analysis

**Figure 10:**
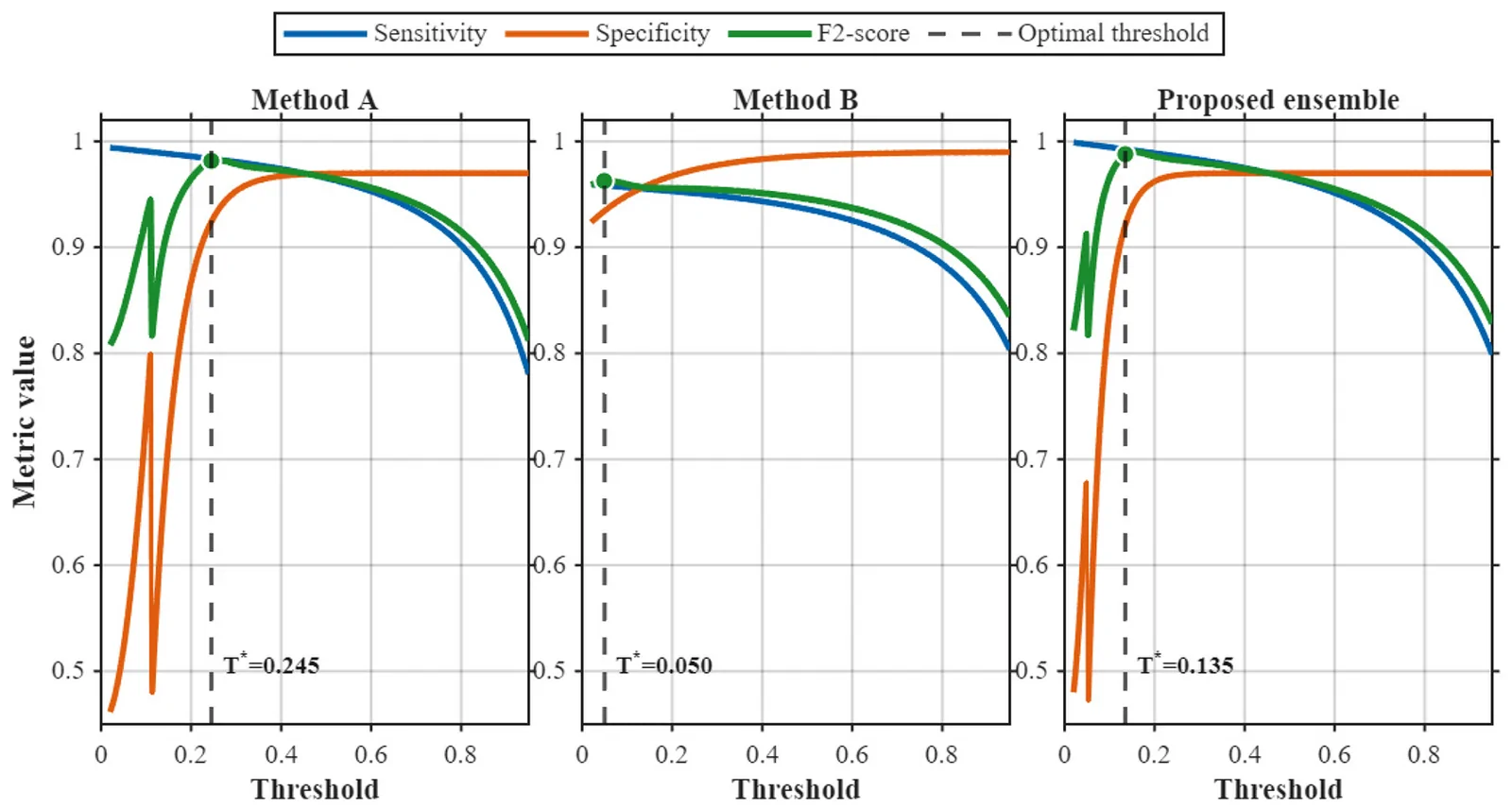
Threshold-optimization behavior of the three evaluated strategies. The selected thresholds are those that best support screening-oriented performance by emphasizing sensitivity and *F*_2_-score.

Figure 10 summarizes the validation-selected operating points. Method A increased sensitivity from 93.03 to 95.84% and reduced false negatives from 144 to 86; Method B increased sensitivity from 91.44 to 97.05% and reduced false negatives from 177 to 61; and the ensemble increased sensitivity from 92.26 to 96.90% and reduced false negatives from 160 to 64. The largest within-model false-negative reduction was 65.54% for Method B. These values quantify threshold effects within each model and do not constitute direct between-model significance tests.

### 4.6 Derived screening indicators and error decomposition

Table 3, Panel B complements accuracy and AUC with NPV, FNR, MCC, sensitivity gain, false-negative reduction, and within-model paired tests. Optimized Method B has the highest NPV (0.9688), the lowest FNR (0.0295), and the largest within-model false-negative reduction (65.54%).

**Figure 11:**
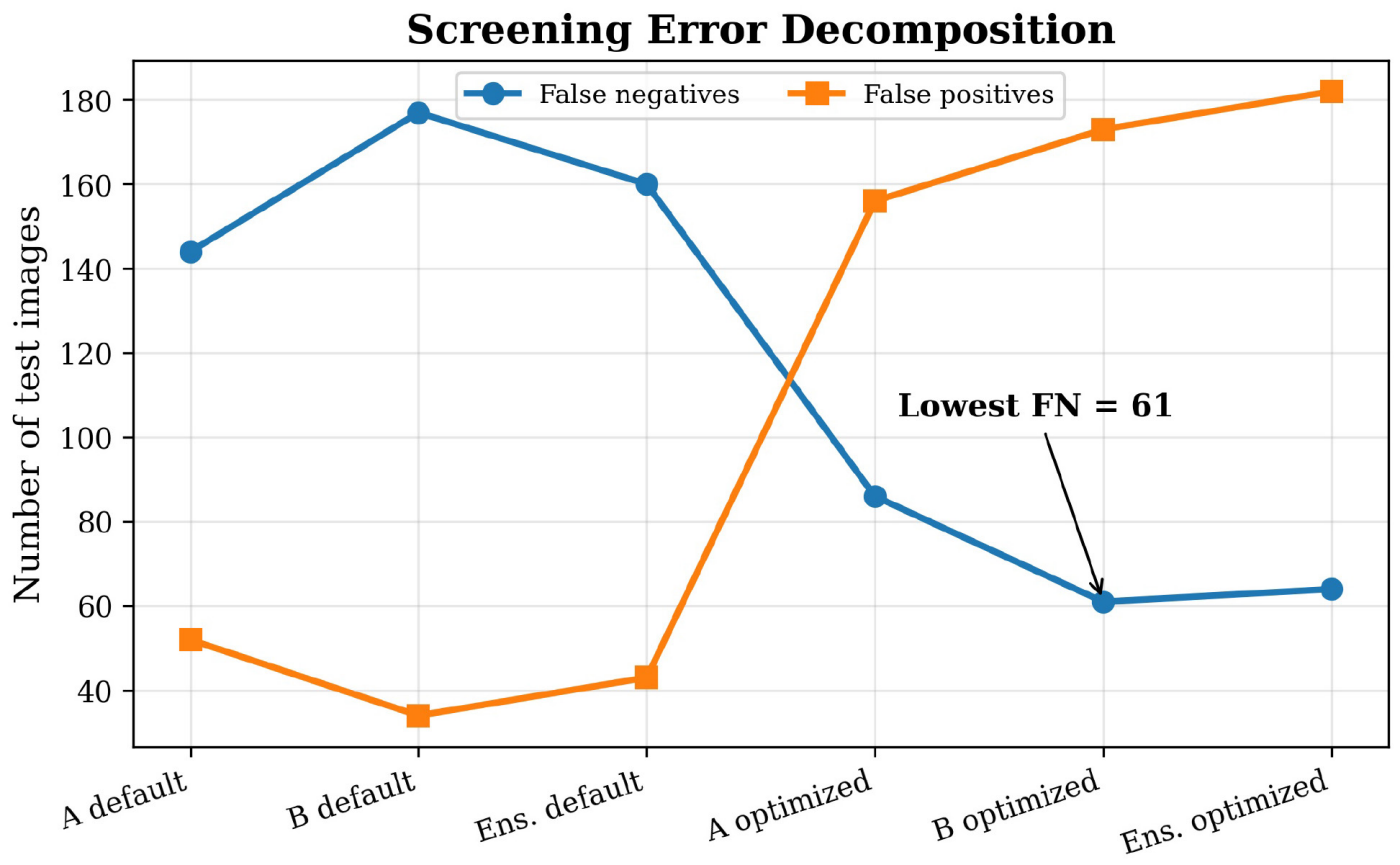
Error decomposition across all evaluated model–decision configurations. The lower false-negative count of Method B optimized is the most important screening advantage revealed by this figure.

**Figure 12:**
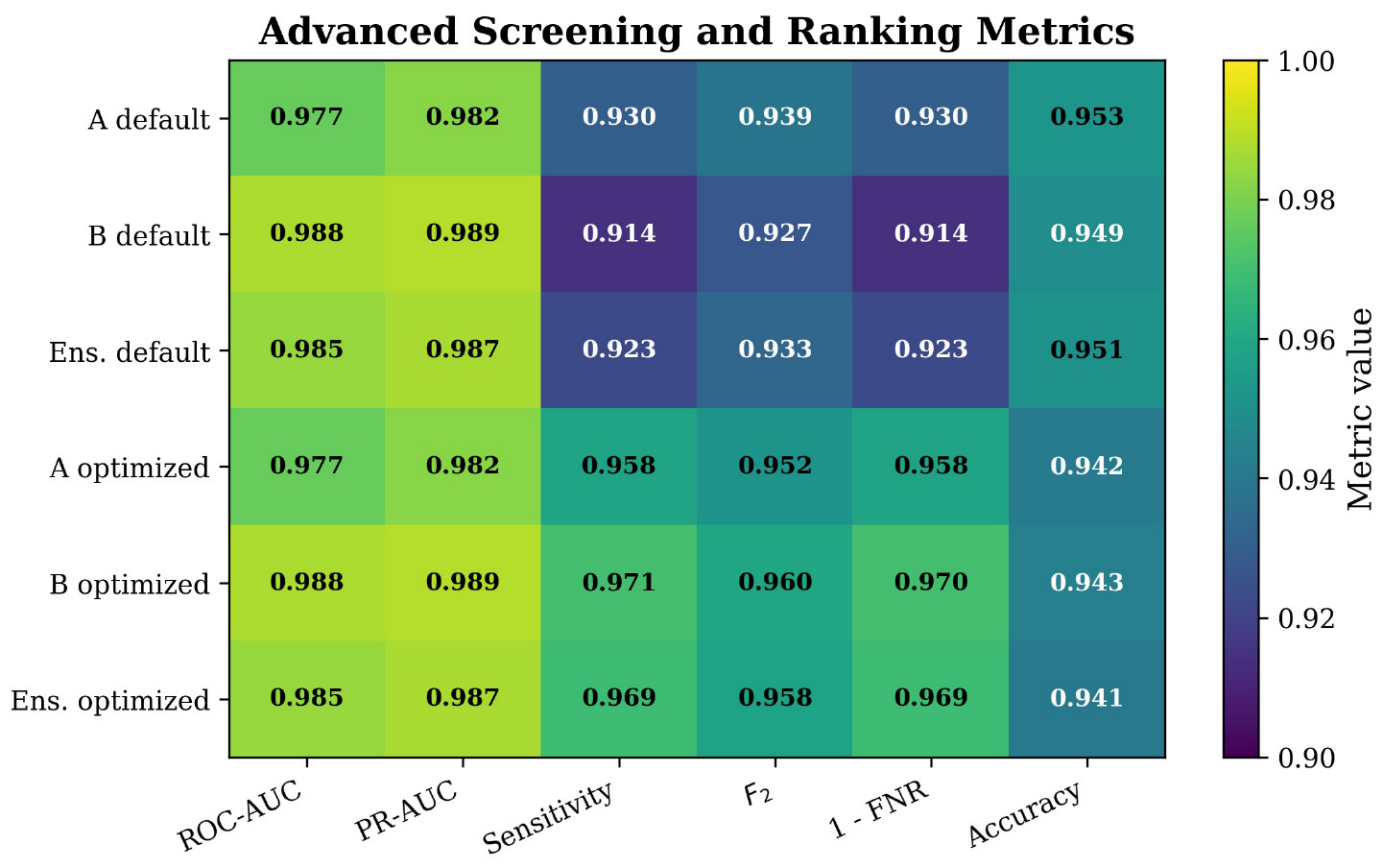
Advanced screening metrics and ranking-oriented indicators. The heatmap shows that different criteria favor different point estimates: Method A at the default threshold has the highest balanced accuracy-oriented values, Method B at the default threshold has the highest AUC-based values, and optimized Method B has the highest sensitivity and *F*_2_ point estimates.

Figure 11 decomposes the screening error-decomposition counts and makes the false-negative advantage of Method B optimized immediately visible. Figure 12 complements this analysis with a heatmap of advanced indicators, showing that Method B optimized dominates the screening-centered metrics, while Method B default dominates the ranking-centered AUC metrics and Method A default remains the strongest in balanced accuracy-oriented behavior.

## 5 Discussion

The principal result is an objective-dependent change in the descriptive ranking. At the conventional threshold, Method A has the highest accuracy-oriented values. After validation-only *F*_2_ optimization, Method B has the highest sensitivity, NPV, and *F*_2_ point estimates and reduces its false negatives from 177 to 61 (65.54%) without retraining. The ensemble produces closely similar screening values, with only three more false negatives than optimized Method B. Aligned per-image outputs required for direct paired between-model testing were not retained in the aggregate analysis files; therefore, this small difference is not interpreted as statistical superiority.

This result also positions the study relative to recent literature. In Mujahid et al. (15), the authors pursued an EfficientNet-oriented malaria classifier, while in Ali et al. (23), the authors emphasized lightweight dilated attention for parasite detection. In Shahin et al. (22), the authors developed a multimodel framework based on feature fusion and voting. In Ahamed et al. (17), the authors strengthened customized CNN models with interpretability tools, while in Parveen et al. (19), the authors expanded trustworthy analysis through explainability and independent-dataset evaluation. The present evidence complements these directions by showing that an additional network or a more elaborate fusion stage is not automatically the best route to safer screening. For the evaluated data, validation-based operating-point selection yields a larger practical reduction in missed parasitized cells than score-level ensembling. This is a meaningful negative result because it identifies where added complexity does not translate into a superior screening trade-off.

These comparisons clarify the practical meaning of our results. The EfficientNet-based study in Marques et al. (26) supports the value of compound-scaled CNNs for malaria-cell recognition. However, architecture-centered approaches do not by themselves answer which operating point should be used when false negatives are clinically more costly. The adversarial-robustness work of Liang and Sun (27) stresses reliability under input perturbation, whereas our experiments examine reliability at the decision-policy level by reducing false negatives through validation-only threshold selection. The attention-based parasite detectors of Zedda et al. (35) are closer to localization and counting, while the acquisition-quality toolkit of Coro et al. (36) addresses the upstream image-quality problem. The present framework therefore fills a downstream gap: once a malaria image classifier produces a probability score, the chosen threshold can determine whether the system behaves as a balanced classifier or as a safer screening assistant. This shifts performance assessment from a static accuracy table to a clinically motivated operating-point analysis.

Fu et al. (24) combined self-supervised learning with attention to strengthen plasmodium detection. Reddy et al. (25) optimized transfer learning by integrating several deep feature extractors. Both studies pursue the same clinical aim: making microscopy-based decision support more dependable. The present results add a decision-policy layer to that aim. Before changing the backbone, expanding the ensemble, or increasing model size, a validation-grounded threshold can already shift the classifier toward the screening behavior that clinicians usually need most–fewer missed positives, with the trade-off stated explicitly.

The figure set summarizes the results within the intended figure limit. The threshold curves show that Method B tolerates aggressive threshold reduction better than the other models. The text-based operating-point summary makes the specificity–sensitivity trade-off explicit without adding another figure: all models gain sensitivity after optimization, but Method B achieves the largest clinically meaningful reduction in missed parasitized cells. The heatmap and integrated dashboard further show that no single metric is sufficient to characterize screening quality. Together, these complementary views describe the screening behavior more completely than a single metric.

### 5.1 Calibration, threshold transferability, and deployment

The optimized thresholds 0.245, 0.050, and 0.135 are dataset-specific operating points rather than universal clinical constants. Changes in microscopy hardware, staining, parasite density, species composition, image quality, or prevalence can shift model scores. Discrimination, probability calibration, and threshold selection should therefore be evaluated separately; a model may retain a high ROC–AUC while its probabilities or a previously selected threshold become unreliable at another site.

For site-level evaluation, the network and preprocessing should first be frozen, followed by patient- and slide-disjoint calibration and test cohorts. Calibration can then be assessed with reliability diagrams, ECE, and the Brier score; any recalibration should be fitted only on the calibration cohort, followed by threshold selection against a prespecified sensitivity target, before one evaluation on the untouched test cohort. Plasmo3Net provides a relevant complementary example through temperature scaling and conformal prediction (46). Calibration statistics are not claimed here because the retained aggregate outputs do not contain the aligned per-image logits required for a defensible *post-hoc* analysis.

### 5.2 Limitations and translational implications

The results are limited to cell-level classification on a curated public dataset. The images are segmented cells rather than complete fields of view, and the public archive does not permit confirmed patient-level or acquisition-site grouping. The image-level split may therefore overestimate generalization if cells from the same patient or slide share acquisition characteristics. No independent external dataset, prospective clinical cohort, low-parasitemia challenge set, or cross-microscope study was available for the present analysis.

The optimized thresholds are dataset-specific and the softmax scores were not prospectively calibrated for a clinical population. Prevalence changes will also alter positive and negative predictive values even when sensitivity and specificity remain stable. Saliency and parasite-like maps are qualitative aids and do not constitute causal explanations. Before clinical translation, the framework requires patient-grouped and site-grouped validation, multicenter external testing, local probability calibration, prospective comparison with expert microscopy, species-aware evaluation, and explicit analysis of low-density infections and staining artifacts. Its current role is research-oriented decision support, not standalone diagnosis.

## 6 Conclusion

This study demonstrates that model selection for malaria-cell screening depends on the operating objective. A balanced set of 27,558 NIH/NLM images was evaluated using a fixed 70:15:15 image-level split, two fully specified custom CNNs, equal-weight score fusion, validation-only *F*_2_ threshold optimization, and confidence-interval analysis. The compact CNN achieved the highest default-threshold accuracy, whereas the deeper CNN achieved the strongest screening-oriented point estimates after threshold optimization: 97.05% sensitivity (95% CI: 96.23%–97.70%), 96.01% *F*_2_, 61 false negatives, and ROC–AUC 0.9876 (95% CI: 0.9842–0.9910). The sensitivity increase relative to the same network at threshold 0.5 was significant (*p* < 0.001). However, the optimized deeper CNN and optimized ensemble have overlapping intervals, so their small difference should not be overstated. The reported performance constitutes internal image-level benchmark evidence only and should not be interpreted as evidence of clinical effectiveness. External patient- and site-disjoint multicenter validation, including cross-microscope and low-parasitemia testing, is required before clinical applicability or transfer of the selected thresholds can be claimed.

## Figures and Tables

**Table:** 

Symbol	Definition
*D*	Complete malaria image collection used in the study
*D*_tr_, *D*_val_, *D*_te_	Training, validation, and independent testing subsets
*N*	Total number of cell images
*x*_*i*_	Input cell image indexed by *i*
*y*_*i*_	Binary class label, with 1 denoting a parasitized cell and 0 denoting an uninfected cell
*I*_*c*_(*u, v*)	Pixel intensity at spatial location (*u, v*) in channel *c*
${\tilde{I}}_{c}\left(u,v\right)$	Normalized image-channel intensity
μ_*c*_, σ_*c*_, ε	Channel mean, channel standard deviation, and stabilizing numerical constant
*M*(*u, v*)	Cell-support mask used to suppress background pixels
*R*(*u, v*)	Parasite-emphasis response map
Φ(·)	Operator combining normalized color and local contrast cues
*p*_*m*_(*x*; θ_*m*_)	Predicted parasitized-cell probability from model *m*
θ_*m*_	Learnable parameters of model *m*
*S*_*A*_, *S*_*B*_, *S*_*ens*_	Scores of Method A, Method B, and the score-level ensemble
α	Ensemble weighting coefficient
τ, τ^⋆^	Candidate and validation-optimized decision thresholds
*T*	Candidate threshold set used during operating-point optimization

## Data Availability

The original contributions presented in the study are included in the article/Supplementary material, further inquiries can be directed to the corresponding author.

## References

[1] World Health Organization. World Malaria Report 2025: Addressing the Threat of Antimalarial Drug Resistance. Geneva: World Health Organization (2025). Available online at: https://www.who.int/publications/i/item/9789240117822 (Accessed August 17, 2026).

[2] LeCun Y Bengio Y Hinton G. Deep learning. Nature. (2015) 521:436–44. doi: 10.1038/nature14539 26017442

[3] Litjens G Kooi T Bejnordi BE Setio AAA Ciompi F Ghafoorian M . A survey on deep learning in medical image analysis. Med Image Anal. (2017) 42:60–88. doi: 10.1016/j.media.2017.07.005 28778026

[4] Shen D Wu G Suk HI. Deep learning in medical image analysis. Annu Rev Biomed Eng. (2017) 19:221–48. doi: 10.1146/annurev-bioeng-071516-044442 28301734 PMC5479722

[5] Simonyan K Zisserman A. Very deep convolutional networks for large-scale image recognition. arXiv. [Preprint]. (2015). arXiv:1409.1556v6. doi: 10.48550/arXiv.1409.1556

[6] He K Zhang X Ren S Sun J. Deep residual learning for image recognition. In: Proceedings of the IEEE conference on computer vision and pattern recognition. Las Vegas, NV: IEEE (2016). p. 770–8. doi: 10.1109/CVPR.2016.90

[7] Szegedy C Vanhoucke V Ioffe S Shlens J Wojna Z. Rethinking the inception architecture for computer vision. In: Proceedings of the IEEE conference on computer vision and pattern recognition. Las Vegas, NV: IEEE (2016). p. 2818–26. doi: 10.1109/CVPR.2016.308

[8] Tan M Le QV. EfficientNet: rethinking model scaling for convolutional neural networks. In: Proceedings of the 36th international conference on machine learning. Long Beach, CA: PMLR (2019). p. 6105–14.

[9] Dosovitskiy A Beyer L Kolesnikov A Weissenborn D Zhai X Unterthiner T . An image is worth 16x16 words: transformers for image recognition at scale. arXiv [Preprint]. (2021). arXiv.2010.11929. doi: 10.48550/arXiv.2010.11929

[10] Rajaraman S Antani SK Poostchi M Silamut K Hossain MA Maude RJ . Pre-trained convolutional neural networks as feature extractors toward improved malaria parasite detection in thin blood smear images. PeerJ. (2018) 6:e4568. doi: 10.7717/peerj.4568 29682411 PMC5907772

[11] Rajaraman S Jaeger S Antani SK. Performance evaluation of deep neural ensembles toward malaria parasite detection in thin-blood smear images. PeerJ. (2019) 7:e6977. doi: 10.7717/peerj.6977 31179181 PMC6544011

[12] Madhu G Mohamed AW Kautish S Shah MA Ali I. Intelligent diagnostic model for malaria parasite detection and classification using imperative inception-based capsule neural networks. Sci Rep. (2023) 13:13377. doi: 10.1038/s41598-023-40317-z 37591916 PMC10435521

[13] Talukdar TR Hossain MJ Talukdar TH. Malaria detection in segmented blood cell using convolutional neural networks and Canny edge detection. arXiv. [Preprint]. arXiv.2202:10426. (2022). doi: 10.48550/arXiv.2202.10426

[14] Huq A Reza MT Hossain S Dipto SM. AnoMalNet: outlier detection based malaria cell image classification method leveraging deep autoencoder. Int J Reconfigur Embed Syst. (2024) 13:171–8. doi: 10.11591/ijres.v13.i1.pp171-178

[15] Mujahid M Rustam F Shafique R Montero EC Alvarado ES de la Torre-Díez I . Efficient deep learning-based approach for malaria detection using red blood cell smears. Sci Rep. (2024) 14:13249. doi: 10.1038/s41598-024-63831-0 38858481 PMC11164904

[16] Hoyos K Hoyos W. Supporting malaria diagnosis using deep learning and data augmentation. Diagnostics. (2024) 14:690. doi: 10.3390/diagnostics14070690 38611603 PMC11012121

[17] Ahamed MF Nahiduzzaman M Mahmud G Shafi FB Ayari MA Khandakar A . Improving malaria diagnosis through interpretable customized CNN architectures. Sci Rep. (2025) 15:6484. doi: 10.1038/s41598-025-90851-1 39987229 PMC11846849

[18] Singh R Prabha C Abdulla S. Optimized CNN framework for malaria detection using Otsu thresholding-based image segmentation. Sci Rep. (2025) 15:40117. doi: 10.1038/s41598-025-23961-5 41249779 PMC12623931

[19] Parveen R Qui B Song W Al-Kahtani N Jamjoom MM Mostafa SM . Trustworthy deep learning for malaria diagnosis using explainable artificial intelligence. Sci Rep. (2025) 15:45037. doi: 10.1038/s41598-025-28387-7 41419508 PMC12748609

[20] Nakasi R Nabende JN Tusubira JF Bamundaga AL Andama A. A dataset of blood slide images for AI-based diagnosis of malaria in Uganda. Data Brief . (2025) 58:111190. doi: 10.1016/j.dib.2024.111190 39802838 PMC11719325

[21] Ramos-Briceño DA Flammia-D'Aleo A Fernández-López G Carrión-Nessi FS Forero-Peña DA. Deep learning-based malaria parasite detection: Convolutional neural networks model for accurate species identification of *Plasmodium falciparum* and *Plasmodium vivax*. Sci Rep. (2025) 15:3746. doi: 10.1038/s41598-025-87979-5 39885248 PMC11782605

[22] Shahin OR Alshammari HH Alabdali RN Salaheldin AM Saleh N. Automated multi-model framework for malaria detection using deep learning and feature fusion. Sci Rep. (2025) 15:25672. doi: 10.1038/s41598-025-04784-w 40664727 PMC12264003

[23] Ali A Pal R Dey I Cuevas E Perez-Cisneros M Sarkar R. DANet: a lightweight dilated attention network for malaria parasite detection. Sci Rep. (2025) 15:36689. doi: 10.1038/s41598-025-20402-1 41120358 PMC12540816

[24] Fu M Wu K Li Y Luo L Huang W Zhang Q. An intelligent detection method for plasmodium based on self-supervised learning and attention mechanism. Front Med. (2023) 10:1117192. doi: 10.3389/fmed.2023.1117192 37457573 PMC10345499

[25] Reddy CKK Anisha PR Almushharaf A Talla R Baili J Cho Y . An optimized transfer learning approach integrating deep convolutional feature extractors for malaria parasite classification in erythrocyte microscopy. Front Med. (2025) 12:1684973. doi: 10.3389/fmed.2025.1684973 41281309 PMC12629934

[26] Marques G Ferreras A de la Torre-Díez I. An ensemble-based approach for automated medical diagnosis of malaria using EfficientNet. Multimed Tools Appl. (2022) 81:28061–78. doi: 10.1007/s11042-022-12624-6 35368860 PMC8964254

[27] Liang L Sun B. Towards an adversarially robust convolutional neural network for automated diagnosis of malaria infection from microscopy images. Biomed Signal Process Control. (2023) 86:105362. doi: 10.1016/j.bspc.2023.105362

[28] Shorten C Khoshgoftaar TM. A survey on image data augmentation for deep learning. J Big Data. (2019) 6:60. doi: 10.1186/s40537-019-0197-0 34306963 PMC8287113

[29] Selvaraju RR Cogswell M Das A Vedantam R Parikh D Batra D. Grad-CAM: Visual explanations from deep networks via gradient-based localization. In: Proceedings of the IEEE international conference on computer vision. Venice: IEEE (2017). p. 618–26. doi: 10.1109/ICCV.2017.74

[30] Ribeiro MT Singh S Guestrin C. Why should I trust you? Explaining the predictions of any classifier. In: *Proceedings of the 22nd ACM SIGKDD international conference on knowledge discovery and data mining*. San Francisco, CA: ACM (2016). p. 1135–44. doi: 10.1145/2939672.2939778

[31] Lundberg SM Lee SI. A unified approach to interpreting model predictions. In: Advances in neural information processing systems.Long Beach, CA: Curran Associates, Inc. (2017). p. 4765–74.

[32] Guo C Pleiss G Sun Y Weinberger KQ. On calibration of modern neural networks. In: Proceedings of the 34th international conference on machine learning. Sydney, Australia: PMLR (2017). p. 1321–30.

[33] Sambyal AS Niyaz U Krishnan NC Bathula DR. Understanding calibration of deep neural networks for medical image classification. Comput Methods Programs Biomed. (2023) 242:107816. doi: 10.1016/j.cmpb.2023.107816 37778139

[34] Gal Y Ghahramani Z. Dropout as a Bayesian approximation: representing model uncertainty in deep learning. In: Proceedings of the 33rd international conference on machine learning. New York, NY: PMLR (2016). p. 1050–9.

[35] Zedda L Loddo A Di Ruberto C. A deep architecture based on attention mechanisms for effective end-to-end detection of early and mature malaria parasites. Biomed Signal Process Control. (2024) 94:106289. doi: 10.1016/j.bspc.2024.106289 39869986

[36] Coro F Mangano V Ahluwalia A De Maria C. Open-source toolkit for image acquisition and quality assessment of thin blood smears for malaria diagnosis. Biomed Signal Process Control. (2025) 103:107470. doi: 10.1016/j.bspc.2024.107470

[37] Alonso-Ramírez AA Barranco-Gutiérrez AI Méndez-Gurrola II Gutiérrez-López M Prado-Olivarez J Pérez-Pinal FJ . Malaria cell image classification using compact deep learning architectures on Jetson TX2. Technologies. (2024) 12:247. doi: 10.3390/technologies12120247

[38] Rubio Maturana C Dantas de Oliveira A Zarzuela F Mediavilla A Martínez-Vallejo P Silgado A . Evaluation of an artificial intelligence-based tool and a universal low-cost robotized microscope for the automated diagnosis of malaria. Int J Environ Res Public Health. (2025) 22:47. doi: 10.3390/ijerph22010047 39857500 PMC11764607

[39] Martín-Ramírez A Lanza-Suárez M Berzosa Díaz P Benito A Antón-Berenguer V Rubio JM. Automated microscopy for malaria diagnosis in a reference laboratory in nonendemic settings. Parasit Vectors. (2026) 19:67. doi: 10.1186/s13071-025-07215-x 41491600 PMC12870222

[40] Xiong H Wang Z Sharan RV Berkovsky S. Uncertainty-guided attention learning for malaria parasite detection in thick blood smears. Neural Netw. (2025) 191:107833. doi: 10.1016/j.neunet.2025.107833 40651249

[41] Sukumarran D Hasikin K Mohd Khairuddin AS Ngui R Wan Sulaiman WY Vythilingam I . Machine and deep learning methods in identifying malaria through microscopic blood smear: a systematic review. Eng Appl Artif Intell. (2024) 133:108529. doi: 10.1016/j.engappai.2024.108529

[42] Dahiya A Raghuvanshi D Sharma C Joshi K Nehra A Sharma A . Deep learning method for malaria parasite evaluation from microscopic blood smear. Artif Intell Med. (2025) 163:103114. doi: 10.1016/j.artmed.2025.103114 40107120

[43] Tiong JY Hasikin K Ngui R Divis PCS Loo CK Lai KW . Insights into AI-driven malaria diagnosis: a systematic review with implications for *Plasmodium knowlesi*. Acta Trop. (2025) 271:107842. doi: 10.1016/j.actatropica.2025.107842 40962162

[44] Faratisha IFD Yunita KC Rahmawati HR Fitri LE Winaris N Muflikah L. Diagnostic accuracy of utilizing artificial intelligence for malaria diagnostic: a systematic review and meta-analysis. Infect Dis Rep. (2026) 18:11. doi: 10.3390/idr18010011 41562666 PMC12821690

[45] XingF LazarevS WooJ. A scoping review of traditional and artificial intelligence methods in malaria diagnostics. NPJ Digit Med. (2026). doi: 10.1038/s41746-026-02880-342298122

[46] Owoloye A Ligali FC Enejoh OA Agosile O Musa AZ Aina O . Plasmo3Net: a convolutional neural network-based algorithm for detecting malaria parasites in thin blood smear images. Next Res. (2026) 11:102093. doi: 10.1016/j.nexres.2026.102093

